# Metagenomics reveals the effects of potassium fertilizer gradients on soil phosphorus-cycling microbial communities and functional genes, and their relationship with yield

**DOI:** 10.3389/fmicb.2026.1918329

**Published:** 2026-09-11

**Authors:** Lele Yang, YiRu Wang, Kexin Zhao, Yixin Ke, Xintong Gao, Enduo Liu, Zhongping Chai

**Affiliations:** 1 College of Resources and Environment, Xinjiang Agricultural University, Urumqi, China; 2 Xinjiang Key Laboratory of Soil and Plant Ecological Processes, Xinjiang Agricultural University, Urumqi, China

**Keywords:** fertilization, metagenomics, pear, soil, yield

## Abstract

The rational application of potassium (*K*) is essential for soil nutrient transformation in orchards. Microbial communities drive phosphorus (*P*) transformation and activation, yet their responses to potassium (*K*) gradients and the underlying regulation of functional potential remain unclear. Accordingly, a field trial was conducted on 7- to 8-year-old Korla pear trees to evaluate changes in microbial community structure and functional potential across topsoil (0–20 cm) and subsoil (40–60 cm) layers under different *K* gradients (0, 75, 150, and 225 kg/ha K_2_O). Results indicated that microbial communities under *K* treatments were distinctly separated from the control. Microbial responses in subsoil were more pronounced than in topsoil, with soil available phosphorus and pH associated with these shifts. At the functional gene level, under K150, microorganisms were characterized by a reduced relative abundance of genes annotated for phosphorus-acquisition-related functions (*ugpE, phy*) and genes associated with nucleotide biosynthesis (*guaA*). With increasing *K* application, the relative abundance of phosphorus-cycling genes in microbial communities exhibited notable differences across *K* gradients. Soil available *K* and microbial biomass nitrogen were closely linked to these functional gene profile variations. Furthermore, *K* fertilization correlated with fruit yield variations, peaking under K150. PLS-PM showed that the community composition of phosphorus-cycling microorganisms and related functional genes had positive relationships with fruit yield. Overall, these results suggest that the K150 treatment corresponded to a favorable balance between fruit yield and microbial genetic potential for nutrient cycling under the conditions of this study.

## 1 Introduction

Pear cultivation imposes stringent demands on soil quality and nutrient management, as pear trees exhibit high sensitivity to mineral availability, particularly potassium (Bosa et al., 2014). As an essential macronutrient, *K* ranks second only to nitrogen in importance and is critical for optimizing fruit yield and quality (Erner et al., 2005). However, the majority of soil potassium (*K*) is sequestered within mineral crystal lattices, which renders it largely inaccessible to plants. Consequently, *K* bioavailability is heavily governed by soil type, physicochemical properties, and other pedogenic factors. To accurately reflect *K* dynamics, soil *K* is typically categorized into four pools based on its availability to plants: water-soluble, exchangeable, non-exchangeable, and structural *K* (Sparks, 1987). Because the vast majority of *K* remains in non-exchangeable and structural forms, a “nutritional paradox” arises where pear trees exhibit *K* deficiency despite growing in *K*-rich soils. Thus, naturally occurring soil *K* pools frequently fail to meet the physiological demands of pear (*Pyrus communis L*.) (Sete et al., 2020). While optimal fertilization is essential for maximizing fruit tree yields, both nutrient deficiencies and surpluses can lead to productivity declines (Ashkevari et al., 2010). In the Korla region of Xinjiang, extensive cultivation and high production intensity have resulted in substantial soil K depletion (Zörb et al., 2014). Despite this, *K* fertilization rates remain generally insufficient, potentially compromising soil *K* supply capacity and inducing nutrient cycling imbalances (Liu et al., 2024), thereby restricting the sustainable development of the pear industry. Unlike annual crop systems, orchards are characterized by deep, perennial root systems that create a unique vertical gradient of nutrient availability. In these systems, phosphorus (*P*) cycling is particularly complex because *P* is relatively immobile in soil, and its availability is heavily dependent on microbial mediation within the rhizosphere. The long-term stability of pear trees makes them highly sensitive to vertical nutrient heterogeneity, yet the mechanisms by which potassium-induced environmental shifts modulate *P*-cycling microbial communities in different soil depths remain poorly understood.

The application of *K* fertilizer alters the soil microenvironment, to which soil microorganisms respond rapidly (Sun et al., 2015). Microbial communities play a pivotal role in maintaining soil fertility and serve as ecological indicators for assessing soil health (Zhu et al., 2020). Microbial community structure is a core metric for measuring ecosystem stability and nutrient turnover efficiency; specifically, variations in alpha and beta diversity reflect the resistance and resilience of soil to environmental perturbations (Pedrinho et al., 2024). *K* fertilizer gradients not only supplement nutrients but also modify the soil habitat, driving the differentiation and recombination of microbial niches. For example, the application of potassium fertilizer directly or indirectly reshapes the evolution of microorganisms by changing physical and chemical properties such as soil pH (Bai et al., 2020). Under such environmental filtering, microbial communities undergo shifts in species composition and their mediated biogeochemical functions. Microorganisms are indispensable to the phosphorus cycle Phosphate-solubilizing microorganisms dissolve insoluble phosphorus by secreting organic acids. This process is regulated by specific genes, such as *gcd*, which chelate metal ions to release phosphate for plant uptake (Rawat et al., 2020). Additionally, organic phosphorus-mineralizing microorganisms synthesize phosphatases (encoded by genes such as *phoD* and *phoA*) to convert organic *P* into available forms (Guo et al., 2023). However, driven by the gradient of potassium fertilizer, the evolution trend of these key microbial groups in the soil of Korla fragrant pear orchard and how the potential of functional genes related to phosphorus cycle responds to the change of potassium level still lack systematic and in-depth analysis (Du et al., 2023).

The Korla pear, with a cultivation history exceeding 1,300 years, is a high-quality specialty of Xinjiang and a protected Geographical Indication product in China (Cheng et al., 2021). Currently, the planting area has reached 62,800 hectares with an annual output of 1.231 million tons (Wang, 2019), making it one of China's vital pear-producing regions. However, orchard management is still dominated by traditional methods; over 90% of orchards utilize flood irrigation, and fertilization is often arbitrary, excessive, or imbalanced. Notably, the over-application of nitrogen combined with insufficient *K* application has led to substantial fertilizer waste, soil degradation, and yield instability. Given the high nutrient demands of Korla pears and their strict quality requirements, optimizing *P*-cycling through precise potassium (*K*) management is essential for sustainable high-yield production. Therefore, to address these gaps, we conducted a metagenomic analysis of soil samples across different depths under varying potassium (*K*) gradients in Korla pear orchards. We hypothesized that: (1) *K* fertilization modulates the soil environment, resulting in significant shifts in the abundance of phosphorus (*P*)-cycling functional genes; (2) *K* fertilization differentially regulates microbial functional potential across soil layers due to the vertical heterogeneity of the pear root system; and (3) an optimal *K* application rate exists that maximizes fruit yield by synchronizing the functional potential of the phosphorus-cycling microbial community. This study provides a theoretical foundation for scientific fertilization and precise soil fertility regulation in Korla pear orchards.

## 2 Materials and methods

### 2.1 Experimental site

The experiment was conducted in Awati Township, Korla, Xinjiang Uygur Autonomous Region, China (42°08′50″N, 86°09′29″E). The study site is located on the northeastern fringe of the Tarim Basin, at the southern foot of the Tianshan Mountains, and adjacent to the northern boundary of the Taklimakan Desert. The regional climate is classified as warm temperate continental arid, characterized by an annual sunshine duration of 2,800–3,100 h, an average annual temperature of 11.0–12.0 °C, and sparse average annual precipitation of approximately 58 mm. Conversely, the annual evaporation rate is high, ranging from 2,700–3,000 mm. Total solar radiation is between 5,600 and 6,400 MJ/m^2^, with an effective accumulated temperature of 4,200–4,500 °C and a frost-free period of 210–240 days. Before the commencement of the experiment, the initial soil physicochemical properties (0–20 cm) were as follows: available nitrogen, 36.09 mg/kg; available phosphorus, 59.67 mg/kg; available potassium, 186.67 mg/kg; organic matter, 18.46 g/kg; and soil pH 7.75.

### 2.2 Experimental design

The research was conducted on 7- to 8-year-old Korla fragrant pear trees. The experiment was carried out in a medium-fertility orchard, applying different rates of potassium fertilizer under drip irrigation. The rootstock used was *Pyrus betulifolia*, with a plant spacing of 1.5 m × 4.0 m and an actual surveyed planting density of 1,500 trees ha^−1^. Throughout the trial, irrigation, nitrogen (*N*), and phosphorus (*P*) application rates were kept constant across all treatments. This standardized fertilization strategy aligns with local orchard management practices and was designed to isolate the effects of potassium (*K*) fertilization on soil microbial communities and phosphorus-cycling functional gene potential. Four levels of potassium fertilizer were applied under drip irrigation, as detailed in Tables 1, 2. While it is important to note that the control treatment (CK) in this study represents a “zero-chemical-potassium” addition rather than an absolute “zero-potassium” treatment, identical baseline *K* was introduced into all plots via the basal sheep manure and irrigation water. Therefore, CK represents the baseline control receiving no supplemental chemical potassium fertilizer, rather than an absolute zero-nutrient condition. Chemical potassium (K_2_O) gradients (0, 75, 150, and 225kg/ha for CK, K75, K150, and K225, respectively) were superimposed on this uniform background. Prior to the experiment, semi-decomposed farmyard sheep manure (containing 18.52% total carbon, 0.76% total nitrogen, 0.52% total phosphorus, and 0.45% total potassium) was applied as a one-time basal fertilizer at a rate of 27,000 kg/ha following the fruit harvest in the previous autumn. The manure was applied via broadcasting and subsequent plowing into the soil. During the growth period of the fragrant pear, nitrogen, phosphorus, and potassium fertilizers were applied. For each treatment, 40% of the nitrogen fertilizer (urea, *N* 46%) was applied before germination, and the remaining 60% was applied at different growth stages. Phosphorus fertilizer (superphosphate, P_2_O_5_ 46%) was applied as a basal fertilizer prior to germination. Potassium fertilizer (potassium sulfate, K_2_O 51%) was applied in two stages, with 40% applied before germination (except in the CK treatment) and the remaining 60% during subsequent growth stages. The basal application of nitrogen, phosphorus, and potassium fertilizers was applied into a ring ditch located 50–80 cm from the trunk, with a width of 30 cm and a depth of 30–60 cm beneath the canopy. The topdressing fertilizer was applied through drip fertigation using a differential pressure fertilization tank with a capacity of 15 L. The fertilizer was dissolved in water the day prior to each application. A 1/4-−1/2-−1/4 irrigation mode was adopted: the first quarter of the irrigation time was used for clear water pre—irrigation, the middle half for fertigation, and the final quarter for flushing the pipeline. All other field management practices were consistent with local agricultural standards.

**Table 1:** Test treatment design scheme.

Experimental treatment	Irrigation requirement (m^3^/ha)	Fertilization scheme N-P_2_O_5_-K_2_O (kg/ha)
CK	7,800	240-240-0
K75	7,800	240-240-75
K150	7,800	240-240-150
K225	7,800	240-240-225

**Table 2:** Irrigation and fertilization system.

Irrigation stage	Irrigation time	Irrigation method	Irrigation frequency	Irrigation water (m^**3**^/ha)		Nutrition level (kg/ha)				
				W	*N*	P_2_O_5_	K_2_O			
							CK	K75	K150	K225
Winter irrigating	20 October	flood irrigation	1	1,800	0	0	0	0	0	0
Spring irrigating	On 15 March	flood irrigation	1	1,800	96	240	0	30	60	90
Fruit bearing periods	5 May	trickle irrigation	1	600	48	0	0	7.5	15	22.5
Early stage of fruit enlargement	25 May	trickle irrigation	1	600	0	0	0	0	0	0
	On 15 June	trickle irrigation	1	600	72	0	0	15	30	45
	5 July	trickle irrigation	1	600	0	0	0	0	0	0
Late fruit expansion	25 July	trickle irrigation	1	600	24	0	0	22.5	45	67.5
	On 15 August	trickle irrigation	1	600	0	0	0	0	0	0
Fruit ripening period	5 September	trickle irrigation	1	600	0	0	0	0	0	0
	Total		9	7,800	240	240	0	75	150	225

### 2.3 Sample collection and analysis

Soil samples were collected at the fruit ripening stage of Korla pears from two soil layers: 0–20 cm and 40–60 cm. The field experiment was conducted within a uniform orchard block (with consistent tree growth and soil conditions), adopting a completely randomized design. The trial was established with four potassium treatments, with three replicate plots set under each treatment. In this study, each plot served as the experimental unit. Each replicate plot consisted of a single row of pear trees. Three pear trees were selected within each experimental unit; these three pear trees served only as sub-samples. Three representative pear trees were randomly selected within each plot; after removing surface litter, soil samples were collected from both sides of the fertilization trenches of these three pear trees and thoroughly mixed to obtain one composite replicate sample for the corresponding soil layer of that plot. Therefore, one composite soil sample collected from the same plot represented a biological replicate at a single soil layer level. Consequently, three replicate samples were obtained for each treatment and each soil layer, resulting in a total of 24 soil samples (4 treatments × 3 plot replicates × 2 soil layers). After initial homogenization and removal of visible debris, samples were sealed in sterile bags and transported to the laboratory in cooling boxes containing dry ice. Upon arrival, samples were screened to remove fine roots and stones. One aliquot was stored at −80°C and subsequently dispatched to Personal Biotechnology Co., Ltd. (Shanghai, China) for shotgun metagenomic sequencing within 7 days. The remaining portion was air-dried and passed through 1 mm and 0.25 mm mesh sieves for subsequent physicochemical characterization. The PLS-PM dataset comprised *N* = 12 independent observations corresponding to the 12 field plots. Since fruit yield is an integrated plot-level metric that cannot be separated by soil depth, soil variables and metagenomic profiles across both depths (0–20 cm and 40–60 cm) were consolidated at the plot level prior to model evaluation, thereby avoiding pseudoreplication by using independent plot-level observations.

The determination of soil physicochemical parameters was performed according to the protocols established by (Bao, 2000). The pH was measured using a pH meter (Mettler-Toledo, Columbus, OH, USA) with a water-to-soil ratio of 2.5:1 (water volume: soil weight). Alkali-hydrolysable nitrogen was determined using the semi-micro Kjeldahl method with a digestion and distillation system (Hanon Instruments, Jinan, China) and reagents (Sinopharm Chemical Reagent Co. Ltd., Shanghai, China). Available phosphorus, available potassium, and organic matter were analyzed using an elemental analyzer (Elementar Analysensysteme GmbH, Langenselbold, Germany), the potassium dichromate oxidation with external heating (Sinopharm Chemical Reagent Co. Ltd., Shanghai, China), the sodium bicarbonate extraction-molybdenum antimony colorimetric method (Sinopharm Chemical Reagent Co. Ltd., Shanghai, China), and the ammonium acetate extraction-flame photometric method (Sinopharm Chemical Reagent Co. Ltd., Shanghai, China), respectively. Soil microbial biomass carbon (MBC) and microbial biomass nitrogen (MBN) were deter-mined by the fumigation-extraction titration method and fumigation-extraction ninhydrin colorimetric method, respectively. A UV-visible spectrophotometer (Shimadzu Corporation, Kyoto, Japan) was applied for colorimetric detection, and all supporting chemicals were purchased from Sinopharm Chemical Reagent Co. Ltd. (Shanghai, China).

### 2.4 Determination of pear yield

In September 2024, at the mature stage of the fragrant pear, a total of 20 fruits per sampled tree (specifically, 5 fruits from the upper and lower branches in each of the four directions: east, south, west, and north) were randomly collected to determine the average single fruit weight. Furthermore, the total number of fruits on each individual replicate tree was recorded. The yield was calculated using the following formula:


$$\begin{array}{r} \text{Yield per plant }=\text{ total number of fruits per plant}\\ \times \text{ single fruitweight}. \end{array}$$

The total yield of 1 ha pear was calculated by the yield per plant.

### 2.5 Metagenomic sequencing

Soil samples (0.5 g each) were ground in liquid nitrogen and total DNA was extracted using the OMEGA Mag-Bind Soil DNA Kit (M5635-02, Omega Bio-Tek, USA) according to the manufacturer's instructions. Extracted DNA was assessed for purity and integrity using a NanoDrop 2000 spectrophotometer (Thermo Fisher Scientific, USA) and 1.2% agarose gel electrophoresis. Qualified samples were stored at −20 °C for subsequent use. Subsequently, DNA was ultrasonically fragmented into approximately 400 bp fragments using a Covaris M220 (Gene Company Limited, China), and libraries were constructed using the NEXTFLEX Rapid DNA-Seq Kit (Bioo Scientific, USA). Following quality control, libraries underwent paired-end sequencing on an Illumina NovaSeq 6000 platform (Personal Biotechnology Co., Ltd., Shanghai, China) (PE150 mode).

To maximize the accuracy of both taxonomic and functional profiling in the complex soil environment of the orchard, we adopted an assembly-based workflow. While taxonomic classification was performed using Kraken2 and Bracken on high-quality reads, functional annotation was conducted on the assembled gene catalog to ensure high-confidence identification of phosphorus-cycling functional genes. Raw sequencing data underwent adapter removal and low-quality base trimming using fastp (Chen et al., 2018), followed by the depletion of host contamination sequences via BMTagger (Rotmistrovsky and Agarwala, 2011). Taxonomic classification of the resulting high-quality reads was initially conducted using Kraken2 (Wood et al., 2019) against the RefSeq database, which encompasses archaea, bacteria, viruses, fungi, and microbial eukaryotes. To rigorously address the potential false-positive classifications typical of complex soil microbiomes, Bracken (Bayesian Reestimation of Abundance with KrakEN; parameter: –*r* 150) was subsequently applied (Steinegger and Söding, 2017). This step statistically re-estimated taxonomic abundances, effectively filtering out spurious, low-confidence assignments. Finally, taxonomic lineages were standardized using TaxonKit (Shen and Ren, 2021), and any reads assigned to multicellular animals or green plants were excluded from downstream analyses. High-quality reads were assembled using MEGAHIT (v1.1.2). The resulting contigs (>300 bp) were clustered using MMseqs2 (easy-linclust mode) at 95% sequence similarity and 90% coverage to construct a non-redundant gene set. The lowest common ancestor taxonomic information for non-redundant contigs was obtained by aligning them against the NCBI-nt database using MMseqs2 (taxonomy mode). Open reading frames (ORFs) were predicted by Prodigal and functionally annotated in KEGG, EggNOG, and CAZy databases using MMseqs2 (search mode). EggNOG and GO classification results were obtained via EggNOG-mapper (v2). GO terms were slimmed using map2slim, and KEGG Orthology (KO) annotations were acquired through KOBAS (Wu et al., 2026). The adopted databases and their corresponding versions/releases were: (1) KEGG release 20200920; (2) eggNOG v5.0.0; (3) CAZy database updated on 07/29/2021. A unified E-value threshold of 1e-5 was applied to filter low-confidence homologous alignments. Additionally, a minimum sequence identity threshold of 70% was required for a hit to be considered valid. For each individual ORF generating multiple significant hits against any database, all matching results were ranked by bit-score in descending order, and only the top 1 hit with the highest bit-score was assigned as the definitive functional annotation of the ORF. To explore potential functional genes responsive to fertilization, an exploratory screening pipeline was applied to the annotated functional profiles. Preliminary statistical screening (*p* < 0.05) was first performed using one-way ANOVA followed by Duncan's multiple range test. Subsequently, a multi-criteria screening strategy was employed with the Benjamini-Hochberg (BH) false discovery rate (FDR) correction: genes satisfying either FDR < 0.1 or nominal significance (*p* < 0.05 and |*log*_2_Ratio > 0.58|) were retained as candidate responsive genes (Supplementary Table S7). All downstream functional gene analyses are presented strictly as exploratory screening results rather than definitive biomarker identifications. These genes (e.g., *guaA, ugpE, htxB*) are frequently reported as key functional components associated with phosphorus-cycling potential across diverse agricultural ecosystems and soil types (Dai et al., 2020; Wang et al., 2024; Ren et al., 2026). This selection ensures that our analysis focuses on representative markers reflecting the broader community-wide shift in genomic functional potential. For details on *Q*-parameter reports (e.g., *Q* > 30) and N50 values for the assembly, see Supplementary Table S5, S6. To quantify functional gene abundances, high-quality reads were mapped back to the non-redundant gene catalog, and read counts were tallied using feature Counts. Crucially, functional gene abundances were calculated using a TPM-like metric (based on the Transcripts Per Kilobase Million formula) to adjust for variations in gene length and sequencing depth across samples. While recent bioinformatic perspectives have highlighted the compositional nature of relative abundance data, TPM-like metrics remain widely adopted in microbial ecology to effectively capture broad, macroscopic shifts in community functional potential. Therefore, to maintain methodological consistency with established ecological literature, these normalized relative abundances were used for downstream comparative analyses.

### 2.6 Statistical analysis

Statistical analyses were executed using IBM SPSS Statistics 26.0 (IBM Corp., Armonk, NY, USA). To evaluate the effects of potassium fertilization treatments on soil microbial communities and functional genes, statistical analyses were performed independently for each soil depth horizon (0–20 cm and 40–60 cm). Pear yield was analyzed separately at the whole-tree level across different treatments. A one-way analysis of variance (ANOVA) followed by Duncan's multiple range test (*p* < 0.05) was applied to identify preliminary differences among potassium levels (CK, K75, K150, and K225). To adjust for multiple hypothesis testing among functionally linked genes, the Benjamini-Hochberg (BH) procedure was applied to raw ANOVA *p*-values. Differentially responsive functional genes were identified using a multi-criteria screening approach: genes meeting either FDR < 0.1 or a combination of nominal *p* < 0.05 and |*log*_2_Ratio > 0.58| were retained. Given the compositional nature of high-throughput sequencing data and the exploratory screening framework adopted here, we acknowledge that these normalization and permissive filtering criteria do not fully resolve compositional-data biases and may carry a risk of inflating gene-level responses. Consequently, all functional gene analyses are presented strictly as an exploratory screening rather than definitive biomarker identification. All graphical visualizations were generated using Origin Pro 2024 (OriginLab Corp., Northampton, MA, USA). Metagenomic data were processed via the GenesCloud platform (Personal Biotechnology Co., Ltd., Shanghai, China), utilizing R(v3.5.1) for diversity and functional profiling. Specifically, Bray-Curtis dissimilarities were calculated using the R package vegan and visualized through Non-metric Multi-dimensional Scaling (Perform Adonis validation). To delineate the associations between functional genes and edaphic factors, Spearman rank correlations were implemented via the ggcorrplot package. The complex direct and indirect associations among potassium inputs, soil properties, functional genes, and crop yield were further evaluated using Partial Least Squares Path Modeling (PLS-PM) in SmartPLS 4 (SmartPLS GmbH, Boenningstedt, Germany). Finally, microbial co-occurrence and gene association networks were constructed based on statistically significant Spearman rank correlations (*r* > 0.6, adjusted *p* < 0.05), with topological parameters and visualizations finalized in Gephi (v0.10.1). Redundancy analysis (RDA) and the envfit test were performed using the cloud computing platform of Personalbio (Shanghai, China). Within this analytical pipeline, the microbial community abundance data were Hellinger-transformed, and all environmental predictors were subjected to *Z*-score standardization prior to model fitting to account for differences in measurement scales.

## 3 Results

### 3.1 Variations in microbial community diversity under different potassium fertilizer gradients

**Figure 1:**
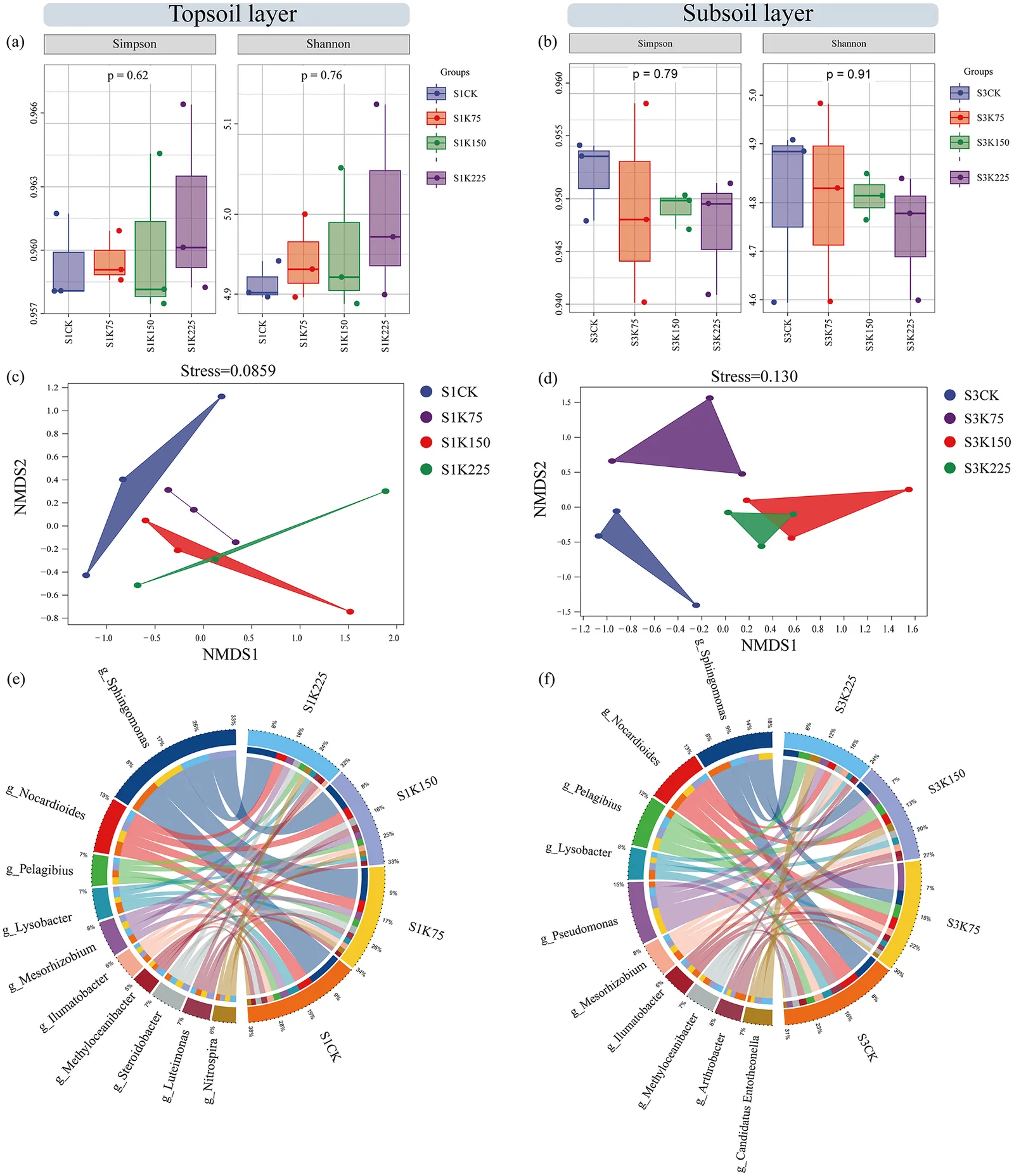
Effects of different potassium fertilizer gradients on soil microbial community diversity and taxonomic composition. **(a, b)** Alpha diversity indices of microbial communities in topsoil (S1, 0–20 cm) and subsoil (S3, 40–60 cm). **(c, d)** Non-metric multidimensional scaling analysis based on Bray-Curtis distance for microbial community structures in surface and subsoil layers. **(e, f)** Chord diagrams illustrating the relative abundance and distribution of the top 10 phosphorus-cycling microbial genera across different potassium treatments in surface and subsoil layers.

No significant differences were observed in the Simpson and Shannon indices across the different soil layers and treatments (Figures 1a, b), indicating that potassium (*K*) fertilization had a minimal impact on soil microbial alpha diversity. To evaluate shifts in the overall microbial community structure, Non-metric Multidimensional Scaling (NMDS) analysis based on Bray-Curtis distances was performed to visualize the community patterns. The stress values were 0.086 and 0.130 for topsoil and subsoil, respectively, indicating reliable ordinations. To formally test the effect of *K* gradients on microbial community structure, a Permutational Multivariate Analysis of Variance was conducted. The results (Supplementary Table S14) indicated that *K* fertilization did not significantly alter community structure in the topsoil layer (*R*^2^ = 0.245, *p* = 0.564), whereas a significant differentiation was observed in the subsoil layer (*R*^2^ = 0.382, *p* = 0.021). Notably, the *P*-cycling microbial communities under the K150 and K225 treatments exhibited high similarity, suggesting that beyond a certain threshold, further increases in *K* application rate have a limited effect on the remodeling of microbial community structure, with the K225 community composition approaching that observed under the K150 treatment. Based on Figures 1e, f, the top 10 phosphorus-cycling microbial genera in terms of relative abundance were identified across different potassium application rates in both surface and subsoil layers. Notably, *Sphingomonas, Nocardioides, Lysobacter*, and *Pseudomonas* emerged as the core dominant taxa across all soil depths.

### 3.2 Shifts in microbial community structure and their correlation with soil nutrients across potassium gradients

**Figure 2:**
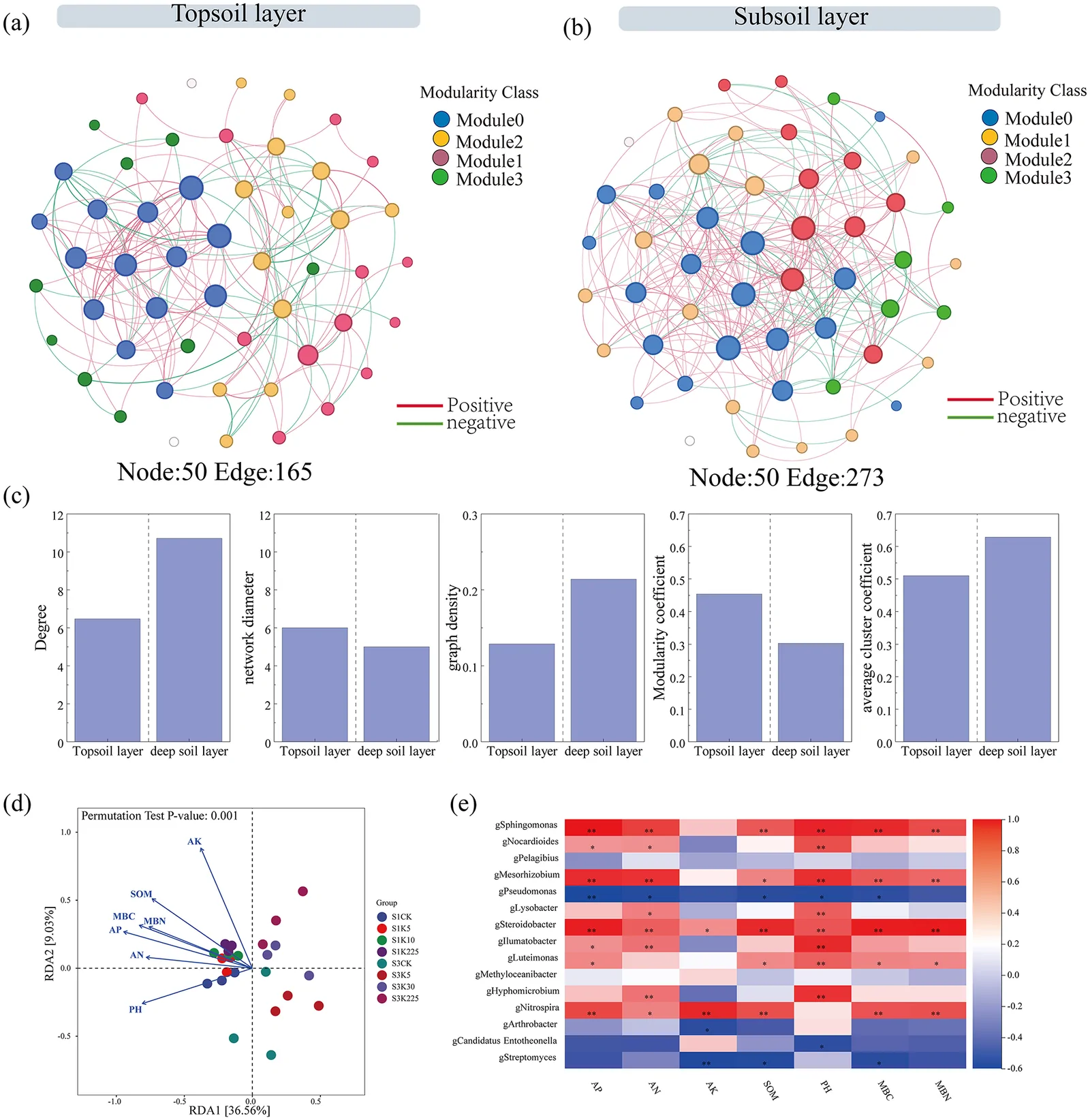
Co-occurrence networks and environmental associations of soil microbial communities in topsoil and subsoil layers under different potassium fertilizer gradients. **(a, b)** Microbial co-occurrence networks in the topsoil and subsoil. **(c)** Comparison of network topological properties between soil layers. **(d)** The relationships between soil environmental factors and microbial community structure. **(e)** Spearman correlation between dominant microbial genera and soil envi-ronmental variables.

To further elucidate the structural patterns of soil phosphorus-cycling microbial communities in the pear orchard, co-occurrence networks were established for both surface and subsoil layers across all treatments based on Spearman correlation analysis (Figures 2a–c). Comparison of network topological indices revealed that the number of edges, average degree, graph density, and average clustering coefficient in the topsoil were lower than those in the subsoil. Conversely, the network diameter and modularity were notably higher in the topsoil. These topological shifts reflect distinct structural configurations between the two soil layers under potassium fertilization. In contrast, subsoil networks exhibited higher connectivity indices, as evidenced by the higher proportion of positive correlations (73.63%) compared to the topsoil (69.09%). These co-occurrence patterns reflect differences in community association structures between the surface and subsoil layers.

In contrast, topsoil networks exhibited distinct modular structures, though whether these topological configurations relate to community stability remains to be tested as a hypothesis. Redundancy analysis coupled with the envfit test was employed to evaluate the relationship between soil physicochemical properties and *P*-cycling microbial community structure (Figure 2d). Permutation tests confirmed the high statistical significance of the RDA model (*p* = 0.001), with the first two canonical axes collectively explaining 44.59% of the total community variation (35.56% by RDA1 and 9.03% by RDA2). The envfit analysis (Supplementary Table S1) further demonstrated that several physicochemical properties contributed significantly to the variations in microbial community structure. Specifically, available phosphorus (*R*^2^ = 0.735, *p* = 0.001), soil pH (*R*^2^ = 0.572, *p* = 0.001), and available nitrogen (*R*^2^ = 0.478, *p* = 0.003) were identified as key explanatory factors. Additionally, microbial biomass carbon (*R*^2^ = 0.591, *p* = 0.001), microbial biomass nitrogen (*R*^2^ = 0.499, *p* = 0.002), and soil organic matter (*R*^2^ = 0.572, *p* = 0.001) also exerted significant influences on the community assembly. However, because the pooled community RDA analyzed topsoil and subsoil samples together without prior variance inflation factor (VIF) filtering, these strongly correlated variables likely represent a composite environmental gradient that is inherently confounded by soil depth. Furthermore, given the limited sample size (*n* = 12 per layer) relative to the numerous predictors fitted in the layer-specific functional-gene RDAs, these models are susceptible to multicollinearity. Therefore, rather than acting as independent drivers, these variables collectively reflect the macro-scale microhabitat differences. The Spearman correlation heatmap further elucidated the specific associations between dominant *P*-cycling genera and soil physicochemical properties. Notably, *Sphingomonas, Mesorhizobium*, and *Nitrospira* exhibited highly significant positive correlations with AP, AN, SOM, and microbial biomass (MBC and MBN). These findings align with the RDA and envfit results, suggesting that these taxa are closely associated with soil phosphorus-related properties. Given their observed distribution patterns, these genera may be linked to soil conditions that favor phosphorus cycling and transformation processes under nutrient-sufficient and optimal pH conditions. Conversely, Pseudomonas showed significant negative correlations with most environmental factors, suggesting a potential competitive disadvantage within the community. Overall, soil available nutrients and pH emerged as the primary factors of shifts in the *P*-cycling microbial community at the genus level.

### 3.3 Variations in phosphorus-cycling functional genes and their correlation with soil nutrients across potassium gradient

**Figure 3:**
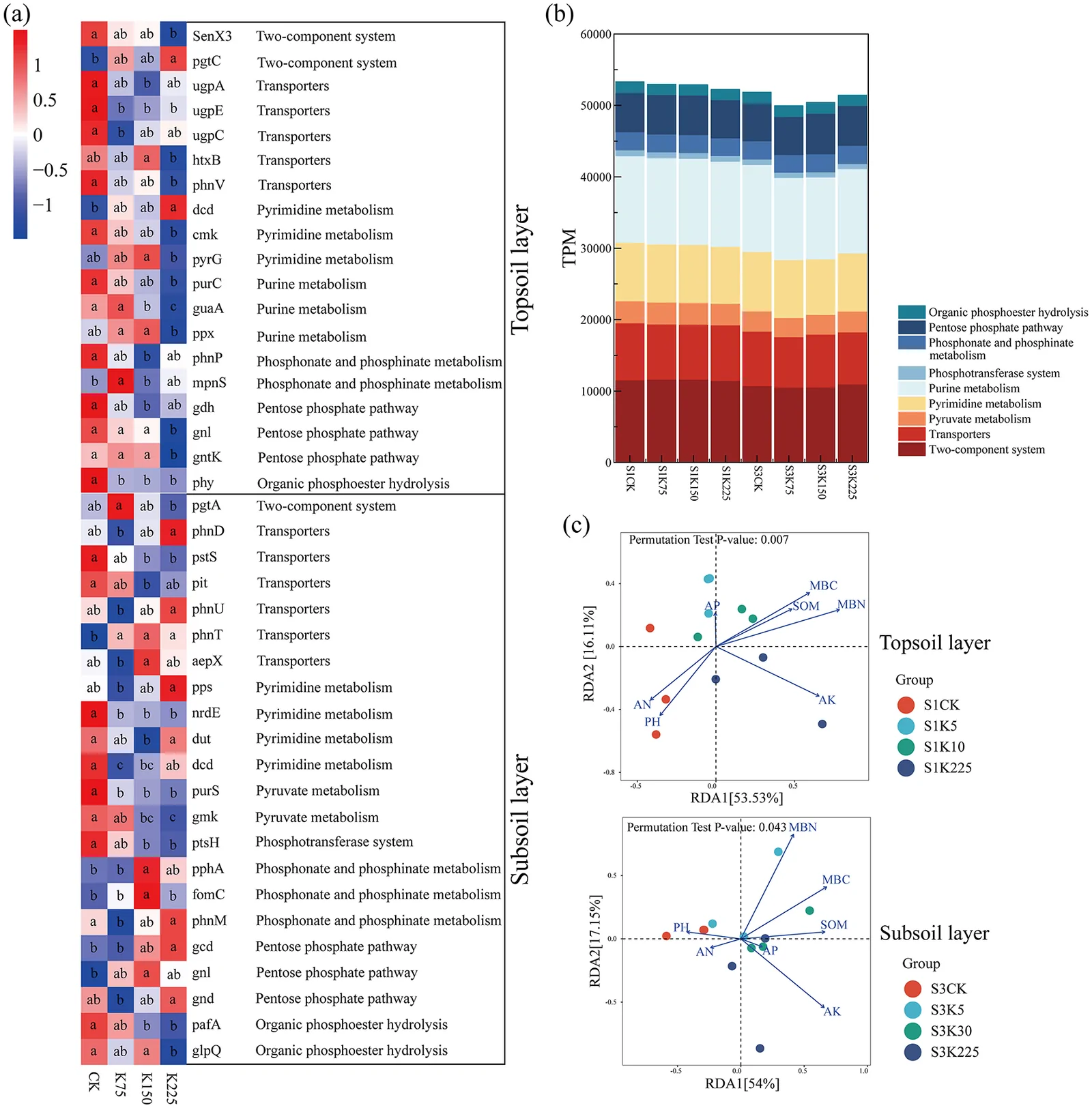
Distribution characteristics of phosphorus **(*P*)** cycling functional genes under different potassium **(*K*)** fertilizer application rates and their relationship with soil physicochemical factors. **(a)** Changes in the abundance of phosphorus cycling functional genes (TPM) in surface and subsoils under different potassium fertilizer application rates. Lowercase letters indicate significant differences among treatments confirmed by one-way analysis of variance (ANOVA) and Duncan's multiple range test (*p* < 0.05). **(b)** Total abundance and composition profiles of phosphorus cycling processes across different treatments and soil layers. **(c)** Redundancy analysis (RDA) illustrating the correlations between *P*-cycling functional genes and soil physicochemical properties.

Metagenomic sequencing results indicated a relative decrease in the genomic potential of phosphorus-cycling genes across fertilization treatments relative to the CK group (Figure 3a). The P-cycling functional genes investigated in this study, along with their detailed functional annotations and KEGG identifiers, are provided in Supplementary Table S12. In the topsoil layer, the relative abundance of phosphorus transporter genes (*ugpA, ugpE, ugpC, phnV*) and genes related to organic phosphoester hydrolysis (*phy*) declined under fertilization treatments. Genes involved in nucleotide biosynthesis (*guaA, purC, cmk, gnl, gntK*) and the two-component system (*SenX3*) followed this downward trend. Deviating from this pattern, *mpnS* increased in the K75 treatment, while *pgtC* and *dcd* increased in the K225 treatment.

In the subsoil layer, fertilization was associated with a relative decrease in the abundance of phosphorus transporters (*pstS, pit*), nucleotide biosynthesis genes (*nrdE, dut, dcd, purS, gmk*), phosphotransferase system genes (*ptsH*), and organic phosphoester hydrolysis genes (*pafA, glpQ*). In contrast, the transporter gene *phnT* increased in abundance across all K treatments compared to CK. Phosphonate functional potential genes (such as *fomC*) and pentose phosphate pathway genes (*gnl, gcd*) also showed increased abundance in the K150 and K225 treatments.

The identified functional genes exhibited consistent preliminary trends across fertilization gradients (Duncan's test, *p* < 0.05; Supplementary Table S7). Further multi-criteria screening was applied as an exploratory screening approach to identify candidate genes meeting either the FDR adjusted threshold (FDR < 0.1) or nominal *p* < 0.05 combined with biological effect sizes (|*log*_2_Ratio| > 0.58; Supplementary Table S7). These candidate genes, categorized by their roles in *P*-cycling, included: phosphorus transport *(ugpE*: FDR < 0.1, *phnT*: |*log*_2_Ratio| > 0.58), organic phosphoester hydrolysis (*glpQ*: FDR < 0.1, *htxB*: |*log*_2_Ratio| > 0.58), and nucleotide biosynthesis (*guaA*: FDR < 0.1, *nrdE*: FDR < 0.1). While the aggregate functional potential of these pathways remained relatively stable, these preliminary candidate features highlight potential community-wide shifts in genomic functional potential under fertilization, bearing in mind the exploratory nature and compositional limitations of these marker-level observations.

To further elucidate the environmental factors associated with shifts in functional genes, redundancy analysis (RDA) coupled with the envfit test was employed to evaluate the relationships between soil physicochemical properties and phosphorus *P*-cycling genes across different soil layers (Figure 3c). Permutation tests indicated that the RDA models were statistically significant for both the topsoil (*p* = 0.007) and subsoil (*p* = 0.043). In the topsoil, the first two canonical axes explained 69.64% of the total variation in functional genes (RDA1: 53.53%; RDA2: 16.11%). Similarly, in the subsoil, the cumulative explanatory power of the first two axes reached 71.15% (RDA1: 54.00%; RDA2: 17.15%). The envfit analysis (Supplementary Table S2) further indicated that multiple physicochemical factors (Supplementary Figure S2) contributed significantly to the variation in phosphorus (*P*)-cycling functional genes. In the topsoil, available potassium (AK; *R*^2^ = 0.46, *p* = 0.05) and microbial biomass nitrogen (MBN; *R*^2^ = 0.63, *p* = 0.01) were identified as the primary factors. Environmental factors exerted a more pronounced influence on microbial community structure in the deep soil layer, where AK (*R*^2^ = 0.69, *p* = 0.003), microbial biomass carbon (MBC; *R*^2^ = 0.52, *p* = 0.028), and MBN (*R*^2^ = 0.62, *p* = 0.008) all exerted significant influences.

Overall, AK emerged as the most critical factor correlated with the *P*-cycling functional gene landscape across both soil layers. Within the RDA ordination space, the vector arrows for microbial biomass indicators (MBC and MBN) and soil organic matter (SOM) formed acute angles and pointed toward the positive direction of the RDA1 axis, indicating a strong positive correlation among these variables. Conversely, soil pH and alkali-hydrolyzable nitro-gen also formed an acute angle with each other but were directed toward the negative RDA1 axis. These results suggest that potassium application is accompanied by concurrent shifts in soil nutrient availability and microbial biomass indicators, which are closely related to the compositional variations of *P*-cycling functional genes.

### 3.4 Effect of fertilization treatment on yield of fragrant pear and PLS structural equation model

#### 3.4.1 Variations in fragrant pear yield across potassium fertilizer gradients

**Figure 4:**
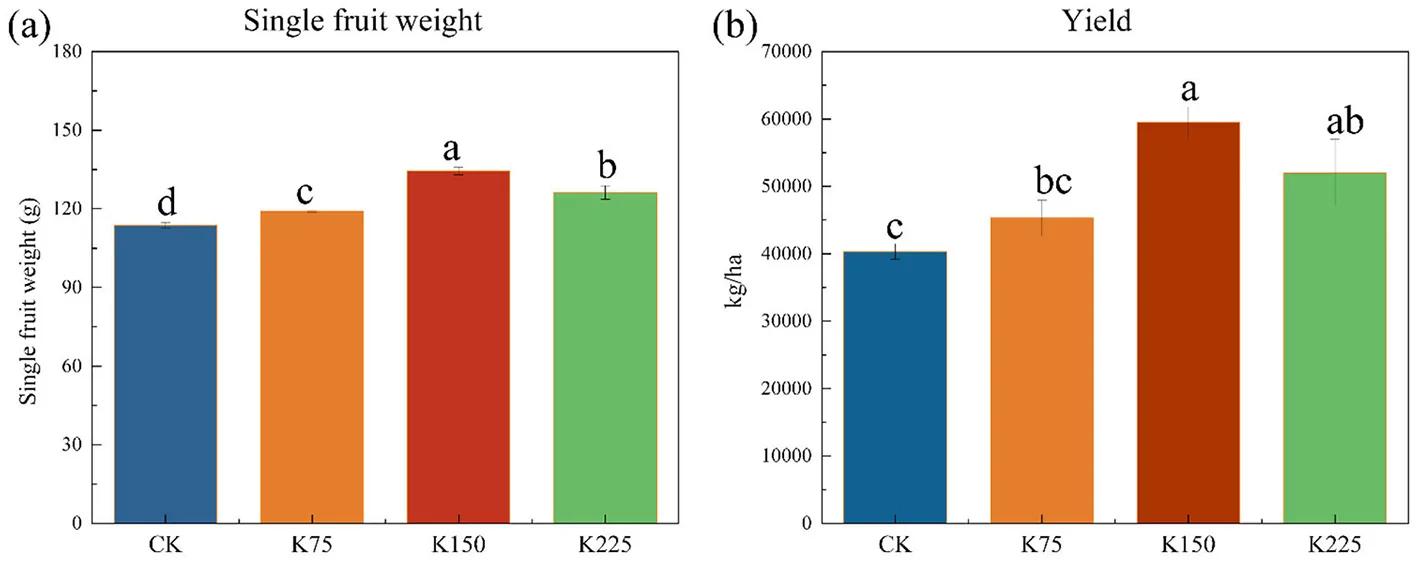
Effects of potassium application rates on single fruit weight **(a)** and yield **(b)**. Different lowercase letters above the bars indicate significant differences between treatments (*p* < 0.05); error bars represent the standard error.

Results indicated that potassium fertilization significantly promoted both pear yield and single fruit weight (Figure 4). With increasing *K* application rates, both metrics followed a significant unimodal trend. To formally validate this trend and identify the optimal application rate, we applied a quadratic regression model. The regression analysis indicated a significant fit for the yield response (*F* = 5.75, *p* = 0.0246, *R*^2^ = 0.56, Supplementary Figure S1), describing a general unimodal trend with the highest observed yield at the 150 kg/ha rate. Beyond this level, yield performance plateaued or declined, suggesting that K150 performed best among the tested gradients under the experimental conditions. Compared to the CK, the K75 treatment significantly increased yield and single fruit weight by 12.24% and 4.67%, respectively (*p* < 0.05). The K150 and K225 treatments exhibited even more substantial gains, with yield and fruit weight increasing by 47.36% and 18.19% for K150, and 28.93% and 10.98% for K225 (*p* < 0.05).

#### 3.4.2 PLS structural equation model

**Figure 5:**
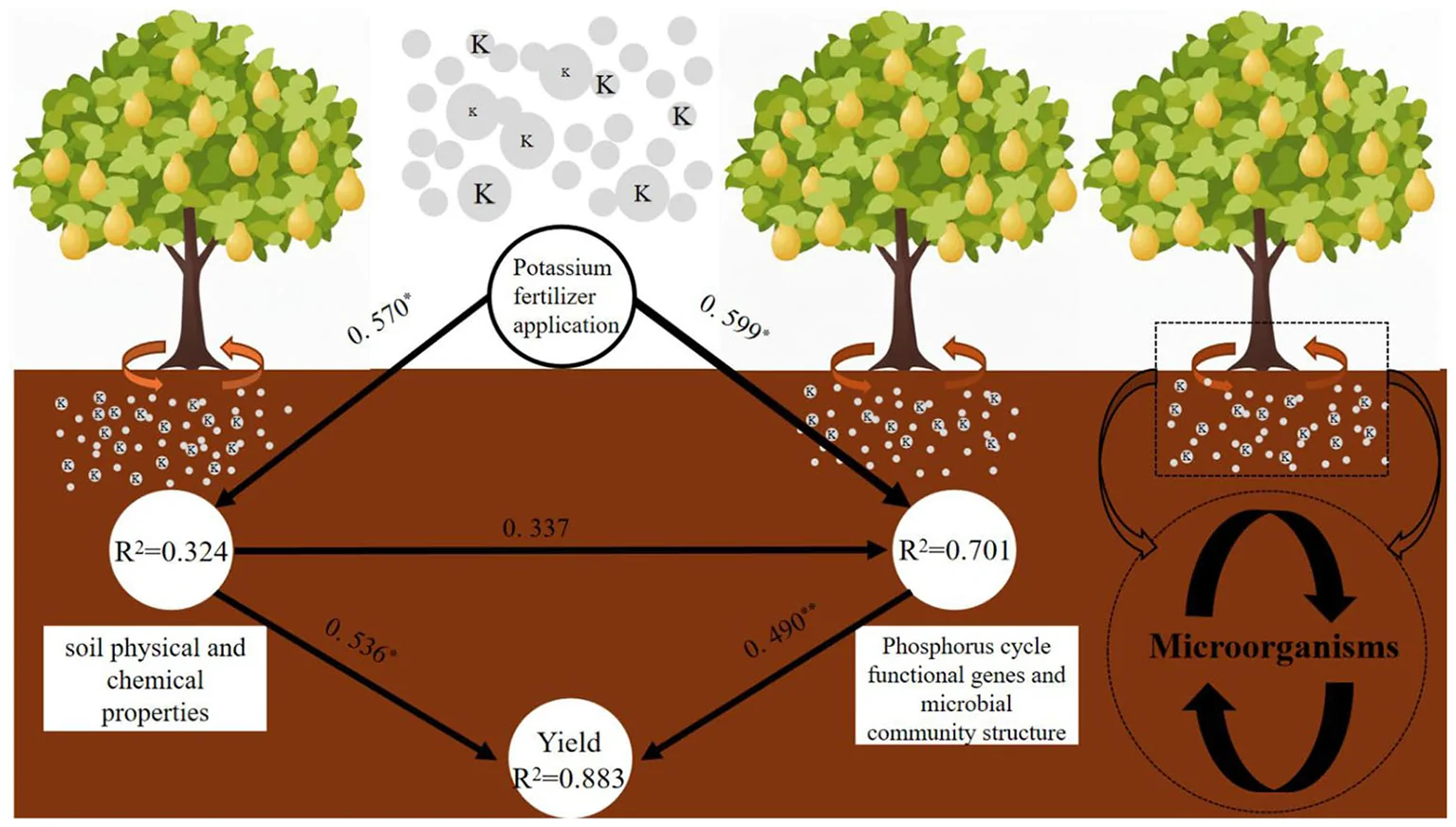
Structural equation model illustrating the effects of potassium fertilization on fruit yield, soil physicochemical properties, and phosphorus cycling. Path descriptions: Solid black arrows represent significant pathways (* is *p* < 0.05, ** is *p* < 0.001), while dashed black arrows represent non-significant pathways. Numbers adjacent to the arrows indicate standardized path coefficients. *R*^2^ values within the circles denote the proportion of variance explained for each latent variable.

A Partial Least Squares Path Model (PLS-PM) was constructed strictly to explore the potential structural associations among potassium (*K*) application, soil properties, *P*-cycling microbial attributes, and fruit yield (Figure 5). The measurement model demonstrated acceptable reliability and validity for most constructs, with quality indicators including composite reliability (rho_c and rho_a), and average variance extracted (AVE) mostly meeting the recommended thresholds. Additionally, the Heterotrait-Monotrait (HTMT) ratios were generally within the acceptable range, supporting the discriminant validity of the latent variables (Supplementary Tables S3,S4, S8–11). The model exhibited an SRMR of 0.185. Given the exploratory nature of this analysis and the sample size constraints (*N* = 12 independent plots), this value is considered acceptable for identifying tentative structural associations. Therefore, all model outputs should be interpreted with appropriate caution regarding sample size and exploratory scope.

The model outcomes showed that the *R*^2^ values for the endogenous constructs soil physical and chemical properties (*R*^2^ = 0.324), *P*-cycling functional genes and microbial communities (*R*^2^ = 0.701), and yield *R*^2^ = 0.883) indicate potential variance trends. Specifically, potassium (*K*) application exhibited positive path coefficients toward *P*-cycling functional genes and microbial communities (path coefficient = 0.599, *p* < 0.05) and soil physicochemical properties (path coefficient = 0.570, *p* < 0.05). Additionally, both microbial *P*-cycling functional genes and microbial communities (path coefficient = 0.490, *p* < 0.05) and soil physicochemical properties (path coefficient = 0.536, *p* < 0.05) were positively associated with fruit yield. Due to sample size constraints, the evaluation of indirect pathways is presented strictly as exploratory estimations. In summary, these results demonstrate clear statistical associations among potassium supply, soil physicochemical properties, microbial communities, *P*-cycling functional gene profiles, and fruit yield.

## 4 Discussion

### 4.1 Variations in microbial community diversity under different potassium fertilizer gradients

The results of this study indicated that potassium fertilizer addition did not significantly alter the alpha-diversity indices of soil microorganisms. However, Adonis analysis revealed a significant differentiation in microbial community structure across different *K* gradients in the subsoil layer (*p* < 0.05), while the topsoil communities showed no such significant response. While alpha-diversity indices provide a coarse snapshot of microbial community characteristics and may not capture subtle shifts in low-abundance taxa, the observed differentiation in beta-diversity suggests a profound structural remodeling. This phenomenon of “stable diversity and structural evolution suggests that *K* fertilizer reshapes the soil microbiome primarily by modulating species composition rather than species richness. It is important to note that changes in community structure (beta-diversity) do not necessarily imply a direct alteration in total functional capacity. Nevertheless, the identified shifts in core dominant genera, such as *Sphingomonas* and *Pseudomonas*, suggest that potassium fertilization may redirect the phosphorus-cycling potential by altering the taxonomic composition of the niche-occupying community (Zhao et al., 2022). Additionally, shifts in phosphorus levels under long-term chemical fertilization have been shown to significantly influence soil microbial diversity (Li et al., 2020; Galanova et al., 2025). Notably, the *P*-cycling microbial communities under the K150 and K225 treatments exhibited a high degree of structural similarity. This observation suggests a potential threshold effect in the response of soil microbiota to potassium enrichment. Specifically, once *K* application reaches a certain level, the environmental selection pressure appears to stabilize, resulting in a weakened driving force for further structural remodeling. This plateauing effect may be attributed to the stabilization of ion interactions and the saturation of microbial nutrient activation pathways at higher *K* concentrations (Yang et al., 2025). Suitable potassium fertilizer can regulate the distribution and transfer of soil microbial assemblages (Song et al., 2025), are associated with variations in the relative abundance of specific microbial groups (Cheng and Wang, 2023). The dominant groups occupy a stable niche, and the community structure may remain stable under the increase of potassium fertilizer application (Chen et al., 2025). *Sphingomonas, Nocardioides, Lysobacter*, and *Pseudomonas* emerged as the core dominant genera across the potassium fertilizer gradients in different soil layers. These microorganisms are known to be actively involved in the mineralization of organic phosphorus and the solubilization of inorganic phosphate (Liao et al., 2021). This suggests that potassium fertilization indirectly regulates phosphorus cycling by altering microbial community composition.

### 4.2 Shifts in microbial community structure and their correlation with soil nutrients across potassium gradients

Co-occurrence network analysis revealed significant divergence in the potential co-occurrence patterns of *P*-cycling microbial communities across different soil layers. The topsoil network was characterized by higher modularity and compartmentalization, whereas the deep-soil network exhibited tighter network linkage. The deep-soil network exhibited higher connectivity (total edges), average degree, and clustering coefficient. Although these topological features show stronger statistical associations among deep-soil microbial taxa, whether such patterns are linked to microbial adaptation to resource-scarce conditions remains hypothetical. These positive correlations may also reflect shared niche preferences or similar physiological responses to the oligotrophic environment (i.e., environmental filtering). Therefore, these patterns should be interpreted as potential co-occurrence associations rather than direct metabolic interactions. We hypothesize that phosphorus-cycling microbial communities in resource-constrained environments might form tighter structural networks, which could potentially be related to nutrient-acquisition-related processes; however, such functional interpretation cannot be directly inferred from our co-occurrence topology (Hu et al., 2006). Conversely, the topsoil network displayed significantly higher modularity. Frequent environmental fluctuations such as fertilization, temperature shifts, and root activity may contribute to this compartmentalized network structure (Thangavel et al., 2022; Ye et al., 2021). Whether such modularity could buffer the community against external disturbances remains speculative and cannot be concluded from the present network dataset. Furthermore, the higher proportion of positive correlations in the deep-soil layer indicates that microbial communities exhibit a highly connected association structure under nutrient-scarce conditions. While this network complexity may reflect underlying ecological associations, it is important to note that co-occurrence patterns can also be driven by shared environmental preferences, common responses to fertilization, or indirect ecological links rather than direct biological interactions. The vertical differentiation in the response of microbial communities to potassium fertilization highlights the complex spatial heterogeneity in orchard ecosystems. Most importantly, this depth-dependent response is highly likely driven by the direct physical placement of basal fertilizers. Because the basal fertilizers were applied in a ring ditch at a depth of 30–60 cm, our deep-soil samples (40–60 cm) directly intersected this localized nutrient hotspot. In contrast, the topsoil (0–20 cm) remained outside the immediate application zone. Furthermore, we hypothesize that this physical fertilization effect may be compounded by the inherent distribution of root systems. In topsoil, high-density root systems generally provide a continuous supply of carbon sources via exudates. We speculate that root-derived carbon supply could support higher functional redundancy and buffering capacity of the surface microbial community, which may contribute to a more stable response to external inputs; nevertheless, these community properties were not directly measured in our study. Conversely, the natural decline in root density in the subsoil limits carbon diffusion. We hypothesize that potassium fertilization directly reshapes the local ionic environment in this inherently resource-constrained zone, and the strengthened network connectivity in deep soil might be associated with nutrient-acquisition-related processes, though this functional link cannot be confirmed solely from co-occurrence data. Future studies incorporating direct measurements of root exudates and micro-scale nutrient dynamics will be necessary to fully disentangle these physical and biological drivers.

The results of redundancy analysis and the *envfit* test were consistent with previous studies (Devau et al., 2011; Liu et al., 2024; Wang et al., 2022), identifying available phosphorus and soil pH as the key factors driving the structural shifts of phosphorus-cycling microbial communities. Furthermore, the Spearman correlation heatmap revealed that dominant *P*-cycling taxa, such as *Sphingomonas*, were significantly and positively correlated with soil nutrient levels. Among these factors, the significant positive correlation with AP indicates that these bacteria may enhance the efficiency of the phosphorus cycle through organic *P* mineralization, phosphate solubilization, and other transformation processes under conditions of improved *P* availability (Heuck et al., 2015; Richardson and Simpson, 2011). SOM, MBC, and MBN serve as key indicators of soil active carbon and nitrogen pools; their correlation with specific genera suggests that sufficient organic substrates and high microbial potential provide the necessary material and energy basis for these functional bacteria (Billings and Ballantyne, 2013). The improvement in overall nutrient availability reshaping the microhabitat not only altered the community structure (Xu et al., 2024) but also increased the relative abundance of dominant functional bacteria involved in the soil *P* cycle (Liu et al., 2024). In summary, potassium fertilization indirectly reshapes the soil microbial community by modulating a covarying suite of available nutrient pools and the soil pH environment across different depth horizons.

### 4.3 Variations in phosphorus-cycling functional genes and their correlation with soil nutrients across potassium gradients

KEGG (Kanehisa et al., 2023), MetaCyc (Caspi et al., 2020), UniProt (Rolf et al., 2004) and other public database information were used to annotate the names and functions of functional genes.

In the topsoil layer, phosphorus-cycling functional genes exhibited distinct compositional variations across the potassium application gradients. Under the K75 treatment, these results suggest that low-level *K* application is associated with a lower relative abundance of genes related to inorganic *P* transport and organic *P* mineralization systems. Previous research has indicated that nutrient fluctuations can trigger a functional redistribution within *P* functional potential (Dai et al., 2020), and *K* fertilization likely modulates this process by altering physical and chemical properties of soil (Spohn and Kuzyakov, 2013; Dai et al., 2020). The enrichment of the *mpnS* gene corresponds to compositional shifts in specific *P*-cycling genes, potentially indicating structural variations in microbial functional profiles under specific potassium application levels (Zhang et al., 2025). Under the K150 treatment, the abundance of *ugpA* and *ugpE* (encoding ABC-type organophosphorus transporters) along with *phy* decline. Concurrently, genes involved in core functional potential processes, such as *guaA* (encoding GMP synthase in purine synthesis) and *gdh* (Glucose/galactose 1-dehydrogenase in the pentose phosphate pathway), also exhibited lower relative abundances. Given that purine metabolism and the pentose phosphate pathway are essential for cell growth and the supply of reducing power (NADPH), as soil nutrient supplies become more balanced or sufficient through potassium fertilization, microorganisms show lower relative abundances of phosphorus acquisition-related genes. This profile likely reflects a lower relative representation of genes associated with high-affinity transport systems, corresponding to shifts in genomic profiles under the improved nutrient environment (Verma et al., 2023; Janssen, 1998). Under the K225 treatment, the abundance of the two-component system gene *SenX3*, which is annotated as encoding a histidine kinase associated with phosphorus limitation sensing pathways, showed a downward trend. This reduction corresponds to a lower relative abundance of genes related to P-limitation pathways in the metagenomic pool, a finding consistent with the decreased abundance of *phy* and *ugp* genes. Additionally, the *pgtC* gene, associated with phosphoglycerol transport and membrane protein, showed shifts that may be associated with compositional variations in the microbial community under high-nutrient conditions (Yan et al., 2023). High-affinity organic *P* acquisition genes, specifically *ugpE* and *phnV*, also exhibited lower relative abundances. This trend likely results from the synergistic effects of nutrient availability (Soumare et al., 2023), which enhance *P* availability (Zheng et al., 2019) and relate to a lower relative presence of transport-related genes. RDA results identified available potassium and microbial biomass nitrogen as the primary environmental factors significantly associated with these patterns, confirming that *K* fertilization is associated with *P*-cycle gene profiles by bolstering carbon and nitrogen pools, consistent with nutrient coupling theories (Dai et al., 2020).

In the K75 treatment of deep soil layers, deep soil metagenomes showed an increased relative abundance of genes related to organic phosphorus acquisition, but a lower relative abundance of genes associated with basic biosynthesis. Subsoil usually has low phosphorus availability (Jalali et al., 2014), and under such conditions, the microbial community composition typically exhibits a higher relative representation of genes associated with utilizing organic phosphorus compounds as alternative phosphorus sources. Potassium fertilizer may alter the deep soil nutrient environment (McEwen and Johnston, 1979), which is accompanied by shifts in microbial community structure (Li et al., 2014) and corresponding variations in the phosphorus acquisition genomic profiles. In the K150 treatment, *P*-transport genes *pstS* and *pit* exhibited lower relative abundances in the subsoil layer. *pstS* is annotated to encode a high-affinity phosphate-binding protein within the Pst ABC transport system, while *pit* is associated with a low-affinity inorganic phosphorus transporter pathway. This lower relative abundance corresponds to a decrease in the relative abundance of genes related to inorganic *P* acquisition. These results indicate variations in microbial phosphorus acquisition profiles under medium potassium application levels. Conversely, genes such as *phnT* (a phosphonate transporter involved in the uptake of *C*—*P* bond compounds) and *fomC* (involved in phosphonate functional potential) showed a significant increase. This divergence indicates a shift in the relative abundance patterns of deep-soil microbiota from inorganic *P*-related genes to organic *P*-related genes. As previously noted, balanced nutrient levels correspond to variations in microbial functional profiles (Soumare et al., 2023), potentially indicating a higher relative representation of genes associated with processing complex organic *P*. This process may be associated with potassium-related variations in microbial community complexity (Liu et al., 2024). At the level of basic biosynthesis pathways, genes related to pyrimidine and purine functional potential showed lower relative abundances, including *nrdE* (encoding ribonucleotide reductase), *dut* (encoding dUTP pyrophosphatase), *dcd* (deoxycytidine deaminase), *purS* (purine synthesis-related enzyme) and *gmk* (guanylate kinase). These enzymes are involved in DNA precursor synthesis and nucleotide biosynthesis, and are the core pathways of cell division and growth. At the same time, *ptsH* (encoding phosphate transfer protein HPr) showed lower relative abundances, which is annotated as being involved in sugar transport. These observations revealed that the community exhibited lower relative abundances of genes associated with rapid growth pathways in the deep environment, but the marked increase of genes related to the pentose phosphate pathway (such as *gnl, gcd*) indicating these compositional shifts in genomic potential. After potassium fertilizer is applied to the subsoil layer, the relative abundance patterns of genes associated with the pentose phosphate pathway show corresponding variations (Gupta and Gupta, 2021). Under the K225 treatment, the abundance of the *pstS* gene (encoding a high-affinity phosphate-binding protein in the Pst ABC system) showed lower relative abundances at the phosphorus transport level. Conversely, the abundance of the transmembrane transport gene *phnT* (encoding a phosphonate transporter involved in *C—P* bond organic phosphonic compound uptake) exhibited a prominent increase. These results suggest a clear shift in genomic potential profiles from inorganic *P* toward alternative pathways associated with organic *P* potential under high-potassium conditions. Furthermore, *ptsH* (encoding phosphate transfer protein HPr) showed lower relative abundances, indicating a lower relative abundance of genes associated with carbon source uptake and phosphate transfer systems. The PTS system is not only involved in glucose functional potential, but also plays a role in carbon source utilization and metabolic (Kotrba et al., 2001). Its decline may coincided with the shift of microbial community from rapid growth to stable functional potential profiles in resource-constrained environments (Litchman et al., 2015). These observations reflect a relative decrease in growth-associated genomic potentials under resource-constrained conditions. Supporting evidence, including the compositional variations of additional key genes, is provided in Supplementary Discussion. The RDA results showed that the first two axes explained 71.15% of the total variation in the subsoil layer, and available potassium was the important environmental variable significantly associated with these patterns. At the same time, MBC was significantly correlated with MBN, indicating that the deep phosphorus cycle genomic profiles were more correlated with potassium level and carbon and nitrogen pool status. pH and AN point to the negative axis, suggesting that soil pH variations may be closely associated with the microbial gene profile shifts in the subsoil.

### 4.4 Effect of fertilization treatment on yield of fragrant pear and PLS structural equation model

Fertilization is the basic means to improve crop productivity. The application of chemical fertilizers can increase crop yield by 55.00%-65.00%, which is an important measure to increase crop yield (Mustafa et al., 2023). Potassium nutrition has become more important in improving the yield of high-yield and high-quality fruits (Kumar and Kavino, 2006). This study shows that pear trees have a significant response to potassium fertilizer. Consistent with previous studies (Hou et al., 2021; Sete et al., 2020; Zeng et al., 2001), the yield and single fruit weight showed a trend of first increase and then decrease with the increase of potassium application rate, with the highest observed values occurring under the K150 treatment. This fertilization level coordinated the nutrient supply well. Although the excessive potassium application was still higher than the control, the yield increase rate exhibited lower relative abundances. It may be related to the decrease of nutrient potential for utilization efficiency and ion antagonism (Rietra et al., 2017).

The PLS-PM analysis provides an exploratory framework to evaluate the structural relationships linking potassium (*K*) fertilization, soil-microbial properties, and fruit yield variations. Overall, yield trends under *K* fertilization correlated closely with shifts in soil physicochemical properties, microbial community composition, and *P*-cycling functional gene profiles. As an essential nutrient, *K* facilitates soil nutrient availability and supports fruit development (Zhao et al., 2010). Furthermore, *K* fertilization was significantly associated with shifts in microbial communities and functional gene profiles, which in turn exhibited strong positive correlations with fruit yield (Lu et al., 2022). It is worth noting that although the K150 treatment exhibited significantly lower relative abundances of certain phosphorus-acquisition-related genes, it corresponded to the highest yield. This may be related to the increased soil available phosphorus content under K fertilization (Supplementary Figure S2), which improved rhizosphere nutrient availability. Crucially, the observed reduction in gene relative abundance represents a shift in genomic potential profiles rather than an actual loss of *in situ* microbial functionality. These PLS-PM associations reflect structural statistical correlations rather than definitive causal mechanisms. Given the experimental sample size (*N* = 12), these findings should be interpreted strictly as an exploratory framework. Under the conditions of this study, the K150 treatment corresponded to distinct configurations of soil nutrient status, microbial community structure, and functional gene features, correlating with the highest observed fruit yield.

## 5 Conclusion

The K150 treatment performed best among the tested rates under the conditions of this study. Potassium input reshapes the microbial phosphorus cycling potential by modulating community structure and *P*-cycling functional genes. Furthermore, *K* fertilization relates to improvements in the soil pH environment and available nutrient status. Analysis indicated that yield variations were closely associated with shifts in microbial community structure and phosphorus-cycling gene abundances. On the one hand, potassium is directly supplied to crops as an essential nutrient element; on the other hand, soil nutrient cycling processes are closely linked to shifts in soil microbial community structure and functional gene abundances. While these findings provide a preliminary basis for *K* management in orchard ecosystems, we acknowledge that this study is based on a single site over one growing season, which may limit the generalizability of our results across different soil types and environmental conditions. Therefore, future research should prioritize long-term field experiments to evaluate the cumulative effects of *K* fertilization, investigate potential interactions with other essential nutrients, and assess the broader applicability of these microbial regulation strategies across diverse soil landscapes.

## 6 Supplementary discussion

Under the K75 treatment in the topsoil layer, genes annotated as high-affinity phosphorus-transport-related genes (ugpE and ugpC) decreased in relative abundance. These genes encode glycerol-3-phosphate ABC transporter subunits, whose annotated functions include the transport of organophosphorus compounds; this pathway has been reported as an important phosphorus-acquisition-related pathway under phosphorus-limited conditions. Concurrently, the relative abundance of *phy* (annotated to encode phytase that catalyzes phytic-acid hydrolysis and inorganic-phosphorus release) also decreased markedly. Conversely, *mpnS*, encoding methyl-phosphonate-synthase-related protein involved in methylphosphonate biosynthesis, showed a significant increase. Under the K225 treatment, nucleotide-synthesis-related genes (*cmk, purC*, and *guaA*) decreased in relative abundance at the genomic-functional-potential level, whereas dcd increased slightly. These compositional shifts within genes associated with DNA-precursor and GMP biosynthesis indicate changes in the genomic functional profile, consistent with previously reported community-level shifts under high-potassium conditions (Li et al., 2023). In addition, the pronounced reduction in the phytase-annotated gene *phy* corresponded to compositional changes in genes linked to organic-phosphorus-hydrolysis-related functions.

Within deep-soil samples under the K75 treatment, the relative abundance of *phnT* increased significantly. This gene encodes a phosphonate transporter annotated as one component of the C-P-related genomic functional system, with predicted functions for transporting organic-phosphorus-containing compounds across cell membranes. Meanwhile, genes annotated to pyrimidine- and purine-related functions (e.g., *nrdE* encoding ribonucleotide reductase; dcd encoding deoxycytidine deaminase; *purS* involved in purine synthesis) decreased in relative abundance. Under the K225 treatment, the relative abundance of pyrimidine- and purine-related genes, including *nrdE* (encoding ribonucleotide reductase, a rate-limiting enzyme annotated for DNA synthesis), *purS*, and *gmk* (annotated for purine synthesis and GMP phosphorylation, respectively), declined markedly. These changes reflect shifts in the composition of genes annotated for DNA-precursor biosynthesis. The pronounced reduction in the organophosphorus-hydrolysis-related genes *pafA* (encoding alkaline phosphatase) and *glpQ* (encoding glycerophosphate diester phosphodiesterase) coincided with altered genomic functional profiles in subsoil samples receiving high potassium inputs.

## Data Availability

The datasets presented in this study can be found in online repositories. The names of the repository/repositories and accession number(s) can be found in the article/Supplementary material.
