# Supplementary material for: Metagenomics reveals the effects of potassium fertilizer gradients on soil phosphorus-cycling microbial communities and functional genes, and their relationship with yield
Source: Front Microbiol. 2026 Sep 11;17:1918329. doi: 10.3389/fmicb.2026.1918329 (PMC13612551; doi:10.3389/fmicb.2026.1918329)
Supplement: Supplementary file 1 [file Supplementary_file_1.docx]

Supplementary

Fig S1. Quadratic regression analysis of the effect of potassium application rates on pear yield.

Fig S2. The Effects of potassium application on soil nutrients in pears orchards

Note: AN = alkali-hydrolyzable nitrogen; AP = available phosphorus; AK = available potassium; SOC = soil organic carbon; pH = soil pH; MBC = microbial biomass carbon; MBN = microbial biomass nitrogen; Different lowercase letters indicate significant differences among treatments (based on one-way ANOVA and Duncan’s multiple range test, *p* < 0.05).

Table S1. Envfit analysis of the relationship between soil physicochemical properties and P-cycling microbial community structure under different potassium fertilizer gradients.

| envfit index | AP | AN | AK | SOM | PH | MBC | MBN |
| --- | --- | --- | --- | --- | --- | --- | --- |
| RDA1 | -0.982 | -0.999 | -0.434 | -0.878 | -0.958 | -0.964 | -0.959 |
| RDA2 | 0.191 | 0.044 | 0.901 | 0.479 | -0.288 | 0.266 | 0.283 |
| R^2^ | 0.735 | 0.478 | 0.597 | 0.572 | 0.572 | 0.591 | 0.499 |
| Pr | 0.001 | 0.003 | 0.001 | 0.001 | 0.001 | 0.001 | 0.002 |

Table S2. Envfit analysis of the relationship between soil physicochemical properties and P-cycling functional gene profiles in topsoil and subsoil layers.

| soil horizon | envfit index | AP | AN | AK | SOM | PH | MBC | MBN |
| --- | --- | --- | --- | --- | --- | --- | --- | --- |
| topsoil layer | RDA1 | 0.030 | -0.810 | 0.935 | 0.907 | -0.689 | 0.883 | 0.958 |
| topsoil layer | RDA2 | 1.000 | -0.587 | -0.354 | 0.422 | -0.725 | 0.469 | 0.288 |
| topsoil layer | R^2^ | 0.037 | 0.268 | 0.464 | 0.274 | 0.288 | 0.444 | 0.636 |
| topsoil layer | Pr | 0.826 | 0.247 | 0.050 | 0.233 | 0.221 | 0.074 | 0.010 |
| deep soil layer | RDA1 | 0.941 | -0.989 | 0.827 | 1.000 | -0.984 | 0.925 | 0.496 |
| deep soil layer | RDA2 | -0.338 | -0.148 | -0.562 | -0.018 | 0.178 | 0.379 | 0.868 |
| deep soil layer | R^2^ | 0.032 | 0.056 | 0.692 | 0.418 | 0.180 | 0.523 | 0.620 |
| deep soil layer | Pr | 0.871 | 0.803 | 0.003 | 0.079 | 0.406 | 0.028 | 0.008 |

Table S3. Partial least squares path model path coefficients

|  | Initial Sample (O) | Sample mean (M) | (STDEV) | T-statistic (\|O/STDEV\|) | Pvalue |
| --- | --- | --- | --- | --- | --- |
| P-cycling functional genes and microbial community -> Yield | 0.49 | 0.487 | 0.174 | 2.826 | 0.005 |
| Physical and Chemical Properties of Soil -> P-cycling functional genes and microbial community | 0.337 | 0.384 | 0.252 | 1.334 | 0.182 |
| Physical and Chemical Properties of Soil -> Yield | 0.536 | 0.52 | 0.207 | 2.587 | 0.01 |
| SF -> P-cycling functional genes and microbial community | 0.599 | 0.57 | 0.245 | 2.447 | 0.014 |
| SF -> Physical and Chemical Properties of Soil | 0.57 | 0.568 | 0.236 | 2.413 | 0.016 |

Table S4. Partial least squares path model (PLS-PM) specific indirect effects path coefficients

|  | Initial Sample (O) | Sample mean (M) | (STDEV) | T-statistic (\|O/STDEV\|) | Pvalue |
| --- | --- | --- | --- | --- | --- |
| SF -> Physical and Chemical Properties of Soil -> P-cycling functional genes and microbial community | 0.192 | 0.218 | 0.192 | 1 | 0.318 |
| Physical and Chemical Properties of Soil -> P-cycling functional genes and microbial community -> Yield | 0.165 | 0.185 | 0.166 | 0.992 | 0.321 |
| SF -> Physical and Chemical Properties of Soil -> Yield | 0.305 | 0.303 | 0.151 | 2.022 | 0.043 |
| SF -> P-cycling functional genes and microbial community -> Yield | 0.294 | 0.282 | 0.144 | 2.04 | 0.041 |
| SF -> Physical and Chemical Properties of Soil -> P-cycling functional genes and microbial community -> Yield | 0.094 | 0.106 | 0.117 | 0.803 | 0.422 |

Table S5. Summary of metagenomic sequencing and assembly statistics.

| 样本名 | Average Sequencing Depth | Total Reads | Contigs | Average Contig Length | Mapping rates |
| --- | --- | --- | --- | --- | --- |
| S1CK1 | 2.51 | 7282749131.00 | 368897.00 | 444.79 | 96.80 |
| S1CK2 | 4.91 | 14263305872.00 | 1024608.00 | 466.76 | 96.96 |
| S1CK3 | 2.70 | 7818852257.00 | 427419.00 | 450.42 | 96.53 |
| S3CK1 | 3.17 | 9208934059.00 | 562214.00 | 452.42 | 97.11 |
| S3CK2 | 3.02 | 8778700830.00 | 448868.00 | 439.56 | 97.07 |
| S3CK3 | 3.26 | 9490078652.00 | 511425.00 | 439.10 | 97.10 |
| S1K751 | 3.20 | 9303591969.00 | 470127.00 | 432.61 | 97.23 |
| S1K752 | 2.81 | 8182370195.00 | 387844.00 | 428.83 | 97.24 |
| S1K753 | 3.60 | 10472245726.00 | 707247.00 | 452.95 | 97.15 |
| S3K751 | 2.63 | 7658455813.00 | 327532.00 | 427.56 | 97.20 |
| S3K752 | 2.80 | 8177446266.00 | 372988.00 | 425.52 | 97.43 |
| S3K753 | 3.20 | 9323051677.00 | 539205.00 | 437.98 | 97.40 |
| S1K1501 | 3.38 | 9845284938.00 | 628846.00 | 484.86 | 97.38 |
| S1K1502 | 3.06 | 8896255960.00 | 683459.00 | 465.51 | 97.17 |
| S1K1503 | 3.31 | 9621439679.00 | 692723.00 | 494.66 | 96.99 |
| S3K1501 | 2.70 | 7874421762.00 | 634113.00 | 472.12 | 97.27 |
| S3K1502 | 2.73 | 7954516090.00 | 572601.00 | 486.61 | 97.42 |
| S3K1503 | 3.05 | 8879010540.00 | 592379.00 | 483.53 | 97.25 |
| S1K2251 | 2.94 | 8584340381.00 | 531594.00 | 459.32 | 97.66 |
| S1K2252 | 2.62 | 7674026826.00 | 424895.00 | 460.72 | 97.70 |
| S1K2253 | 2.88 | 8391433777.00 | 718468.00 | 488.96 | 97.30 |
| S3K2251 | 4.84 | 14097302644.00 | 911955.00 | 467.35 | 97.30 |
| S3K2252 | 3.84 | 11227916840.00 | 868481.00 | 458.31 | 97.58 |
| S3K2253 | 3.43 | 9985826408.00 | 643101.00 | 450.45 | 97.20 |

Table S6. Quality control metrics for raw metagenomic sequencing data.

| 样本名 | 测得Reads总数 | 测得碱基总数(bp) | N (%) | GC (%) | Q20 (%) | Q30 (%) | N50 |
| --- | --- | --- | --- | --- | --- | --- | --- |
| S1CK1 | 49892616 | 7533785016 | 0.0025 | 64.93 | 98.61 | 95.98 | 654 |
| S1CK2 | 97547768 | 14729712968 | 0.0025 | 64.26 | 98.66 | 96.12 | 741 |
| S1CK3 | 53700158 | 8108723858 | 0.0025 | 64.24 | 98.58 | 95.91 | 689 |
| S3CK1 | 67082178 | 10129408878 | 0.0025 | 64.23 | 98.71 | 96.3 | 908 |
| S3CK2 | 60709190 | 9167087690 | 0.0025 | 64.85 | 98.68 | 96.24 | 792 |
| S3CK3 | 65813796 | 9937883196 | 0.0025 | 65.05 | 98.64 | 96.14 | 944 |
| S1K751 | 62885286 | 9495678186 | 0.0026 | 64.75 | 98.77 | 96.44 | 693 |
| S1K752 | 59942926 | 9051381826 | 0.0026 | 64.19 | 98.72 | 96.27 | 651 |
| S1K753 | 64802626 | 9785196526 | 0.0026 | 64.35 | 98.66 | 96.16 | 656 |
| S3K751 | 53721022 | 8111874322 | 0.0025 | 63.83 | 98.74 | 96.38 | 864 |
| S3K752 | 54183188 | 8181661388 | 0.0034 | 64.11 | 98.92 | 96.89 | 962 |
| S3K753 | 60579614 | 9147521714 | 0.0026 | 64.02 | 98.77 | 96.47 | 881 |
| S1K1501 | 63479002 | 9585329302 | 0.0025 | 63.81 | 98.7 | 96.28 | 611 |
| S1K1502 | 55806984 | 8426854584 | 0.0025 | 64.15 | 98.74 | 96.36 | 617 |
| S1K1503 | 71507542 | 10797638842 | 0.0027 | 64.39 | 98.69 | 96.22 | 704 |
| S3K1501 | 58316346 | 8805768246 | 0.0081 | 63.27 | 98.84 | 96.66 | 763 |
| S3K1502 | 52141394 | 7873350494 | 0.0079 | 63 | 98.82 | 96.64 | 800 |
| S3K1503 | 57232362 | 8642086662 | 0.0081 | 63.54 | 98.68 | 96.27 | 973 |
| S1K2251 | 52255044 | 7890511644 | 0.0082 | 63.45 | 98.66 | 96.2 | 611 |
| S1K2252 | 55686986 | 8408734886 | 0.0082 | 63.38 | 98.69 | 96.3 | 615 |
| S1K2253 | 63482182 | 9585809482 | 0.0082 | 63.99 | 98.76 | 96.46 | 656 |
| S3K2251 | 96134632 | 14516329432 | 0.0082 | 63.48 | 98.66 | 96.22 | 822 |
| S3K2252 | 76339202 | 11527219502 | 0.0083 | 63.25 | 98.87 | 96.76 | 773 |
| S3K2253 | 68129858 | 10287608558 | 0.0082 | 63.8 | 98.7 | 96.3 | 719 |

Table S7-1. Statistical information of phosphorus cycling functional genes screened by nominal p < 0.05 and |log₂Ratio| > 0.58 criteria

| FC | Gene | Treatment_Contrast | Direction | log2Ratio_EffectSize | \|log_2Ratio\| > 0.58 | Uncertainty_SE |
| --- | --- | --- | --- | --- | --- | --- |
| Topsoil | htxB | S1K10 vs S1CK | Enriched | 0.093 | 2.063 | 0.1491 |
|  | htxB | S1K15 vs S1CK | Depleted | -2.063 |  | 0.1291 |
|  | htxB | S1K5 vs S1CK | Depleted | -0.75 |  | 0.2344 |
| Topsoil | pgtC | S1K10 vs S1CK | Enriched | 0.204 | 0.599 | 0.5117 |
|  | pgtC | S1K15 vs S1CK | Enriched | 0.6 |  | 0.483 |
|  | pgtC | S1K5 vs S1CK | Enriched | 0.466 |  | 1.0802 |
| Topsoil | mpnS | S1K10 vs S1CK | Depleted | -1.28 | 3.037 | 0.0421 |
|  | mpnS | S1K15 vs S1CK | Enriched | 1.48 |  | 0.2855 |
|  | mpnS | S1K5 vs S1CK | Enriched | 3.037 |  | 0.2096 |
| Subsoil | htxA | S3K10 vs S3CK | Enriched | 0.892 | 0.8919 | 1.6424 |
|  | htxA | S3K15 vs S3CK | Enriched | 0.486 |  | 1.8327 |
|  | htxA | S3K5 vs S3CK | Depleted | -0.201 |  | 0.9205 |
| Subsoil | nrdE | S3K10 vs S3CK | Depleted | -0.932 | 1.0364 | 0.4245 |
|  | nrdE | S3K15 vs S3CK | Depleted | -1.036 |  | 1.7284 |
|  | nrdE | S3K5 vs S3CK | Depleted | -0.88 |  | 2.5598 |
| Subsoil | fomC | S3K10 vs S3CK | Enriched | 0.606 | 0.6058 | 0.5491 |
|  | fomC | S3K15 vs S3CK | Enriched | 0.119 |  | 0.7584 |
|  | fomC | S3K5 vs S3CK | Enriched | 0.248 |  | 0.3613 |
| Subsoil | pafA | S3K10 vs S3CK | Depleted | -0.54 | 0.6909 | 1.3446 |
|  | pafA | S3K15 vs S3CK | Depleted | -0.691 |  | 0.3781 |
|  | pafA | S3K5 vs S3CK | Depleted | -0.152 |  | 1.9136 |
| Subsoil | phnT | S3K10 vs S3CK | Enriched | 0.99 | 0.9898 | 0.2383 |
|  | phnT | S3K15 vs S3CK | Enriched | 0.747 |  | 0.5609 |
|  | phnT | S3K5 vs S3CK | Enriched | 0.822 |  | 0.3533 |
| Subsoil | aepX | S3K10 vs S3CK | Enriched | 0.555 | 0.9006 | 1.8878 |
|  | aepX | S3K15 vs S3CK | Enriched | 0.071 |  | 0.2707 |
|  | aepX | S3K5 vs S3CK | Depleted | -0.901 |  | 0.8977 |
| Subsoil | pphA | S3K10 vs S3CK | Enriched | 0.974 | 0.9734 | 0.4328 |
|  | pphA | S3K15 vs S3CK | Enriched | 0.566 |  | 0.904 |
|  | pphA | S3K5 vs S3CK | Depleted | -0.033 |  | 0.2082 |

Table S7-2. Statistical information of phosphorus cycling functional genes screened by FDR < 0.1 criterion

| FDR | Gene | Treatment_Contrast | Direction | Nominal_p_value | Adjusted_p_value_FDR | Uncertainty_SE |
| --- | --- | --- | --- | --- | --- | --- |
| Topsoil | guaA | S1K10 vs S1CK | Depleted | 0.001504 | 0.006016 | 2.8248 |
|  |  | S1K15 vs S1CK | Depleted |  |  | 10.0735 |
|  |  | S1K5 vs S1CK | Enriched |  |  | 3.6666 |
| Topsoil | gnl | S1K10 vs S1CK | Depleted | 0.006358 | 0.01272 | 7.6172 |
|  |  | S1K15 vs S1CK | Depleted |  |  | 6.6829 |
|  |  | S1K5 vs S1CK | Depleted |  |  | 10.4047 |
| Topsoil | ugpE | S1K10 vs S1CK | Depleted | 0.01149 | 0.01533 | 9.8972 |
|  |  | S1K15 vs S1CK | Depleted |  |  | 2.3697 |
|  |  | S1K5 vs S1CK | Depleted |  |  | 13.7722 |
| Topsoil | gntK | S1K10 vs S1CK | Enriched | 0.01791 | 0.01791 | 5.6162 |
|  |  | S1K15 vs S1CK | Depleted |  |  | 13.4386 |
|  |  | S1K5 vs S1CK | Enriched |  |  | 8.2789 |
| Subsoil | glpQ | S3K10 vs S3CK | Depleted | 0.01398 | 0.01596 | 3.5486 |
|  |  | S3K15 vs S3CK | Depleted |  |  | 12.5496 |
|  |  | S3K5 vs S3CK | Depleted |  |  | 3.7582 |
| Subsoil | dcd | S3K10 vs S3CK | Depleted | 0.01474 | 0.01596 | 7.4873 |
|  |  | S3K15 vs S3CK | Depleted |  |  | 4.4529 |
|  |  | S3K5 vs S3CK | Depleted |  |  | 2.0242 |
| Subsoil | fomC | S3K10 vs S3CK | Enriched | 0.01596 | 0.01596 | 0.5493 |
|  |  | S3K15 vs S3CK | Enriched |  |  | 0.7584 |
|  |  | S3K5 vs S3CK | Enriched |  |  | 0.3614 |
| Subsoil | phnT | S3K10 vs S3CK | Enriched | 0.01345 | 0.01596 | 0.2383 |
|  |  | S3K15 vs S3CK | Enriched |  |  | 0.5609 |
|  |  | S3K5 vs S3CK | Enriched |  |  | 0.3534 |

Table S8. Phosphorus Cycle Partial Least Squares Path Model Quality Standard Fit Index: AVE

| AVE | Initial Sample (O) | Sample mean (M) | STDEV | T-statistic (O/STDEV) | Pvalue |
| --- | --- | --- | --- | --- | --- |
| P-cycling functional genes and microbial community | 0.39 | 0.454 | 0.079 | 4.954 | 0 |
| Physical and Chemical Properties of Soil | 0.736 | 0.726 | 0.095 | 7.739 | 0 |
| SF | 1 | 1 | 0 | n/a | n/a |
| Yield | 0.776 | 0.779 | 0.081 | 9.577 | 0 |

Table S9. Phosphorus Cycle Partial Least Squares Path Model Quality Standard Fit Index: rho_c

| rho_c | Initial Sample (O) | Sample mean (M) | STDEV | T-statistic (O/STDEV) | Pvalue |
| --- | --- | --- | --- | --- | --- |
| P-cycling functional genes and microbial community | 0.507 | 0.486 | 0.159 | 3.18 | 0.001 |
| Physical and Chemical Properties of Soil | 0.571 | 0.568 | 0.152 | 3.749 | 0 |
| SF | 1 | 1 | 0 | n/a | n/a |
| Yield | 0.912 | 0.91 | 0.045 | 20.262 | 0 |

Table S10. Phosphorus Cycle Partial Least Squares Path Model Quality Standard Fit Index: rho_a

| rho_a | Initial Sample (O) | Sample mean (M) | STDEV | T-statistic (O/STDEV) | Pvalue |
| --- | --- | --- | --- | --- | --- |
| P-cycling functional genes and microbial community | 0.891 | 0.904 | 0.034 | 26.392 | 0 |
| Physical and Chemical Properties of Soil | 0.878 | 0.893 | 0.128 | 6.861 | 0 |
| SF | 1 | 1 | 0 | n/a | n/a |
| Yield | 0.884 | 0.888 | 0.055 | 15.97 | 0 |

Table S11. Phosphorus Cycle Partial Least Squares Path Model Quality Standard Fit Index: HTMT

| HTMT | Initial Sample (O) | Sample mean (M) | 2.5% | 97.5% |
| --- | --- | --- | --- | --- |
| Physical and Chemical Properties of Soil <-> P-cycling functional genes and microbial community | 0.685 | 0.789 | 0.546 | 1.04 |
| SF <-> P-cycling functional genes and microbial community | 0.73 | 0.749 | 0.58 | 0.924 |
| SF <-> Physical and Chemical Properties of Soil | 0.616 | 0.64 | 0.252 | 0.966 |
| Yield <-> P-cycling functional genes and microbial community | 0.817 | 0.864 | 0.693 | 1.106 |
| Yield <-> Physical and Chemical Properties of Soil | 1.011 | 1.021 | 0.866 | 1.217 |
| Yield <-> SF | 0.793 | 0.791 | 0.482 | 1.016 |

Table 12. Functional classification and KEGG orthology (KO) information of phosphorus-cycling related genes.

| Gene | KO | Pathway | Functional Role |
| --- | --- | --- | --- |
| SenX3 | K07768 | Two-component system | Sensor histidine kinase SenX3 (OmpR family) |
| cmk | K00945 | Pyrimidine metabolism | Cytidylate kinase(Central pyrimidine metabolism housekeeping gene) |
| dcd | K01494 | Pyrimidine metabolism | dCTP deaminase(Central pyrimidine metabolism housekeeping gene) |
| gdh | K00034 | Pentose phosphate pathway | Glucose/galactose 1-dehydrogenase(Central carbohydrate metabolism gene) |
| gnl | K01053 | Pentose phosphate pathway | Gluconolactonase(Central pentose-phosphate pathway gene) |
| gntK | K00851 | Pentose phosphate pathway | Gluconokinase(Central pentose-phosphate pathway gene) |
| guaA | [K01951](https://www.genome.jp/entry/K01951) | Purine metabolism | GMP synthase (glutamine-hydrolysing)(Central purine metabolism housekeeping gene) |
| htxB | K02044 | Transporters | Hypophosphite transport system substrate-binding protein |
| mpnS | K18049 | Phosphonate and phosphinate biosynthesis | Methylphosphonate synthase |
| pgtC | [K08478](https://www.genome.jp/entry/K08478) | Two-component system | Phosphoglycerate transport regulatory protein PgtC |
| phnP | K06167 | Phosphonate and phosphinate metabolism | Phosphoribosyl 1,2-cyclic phosphate phosphodiesterase |
| phnV | K11082 | Transporters | 2-aminoethylphosphonate transport system permease |
| phy | [K22906](https://www.genome.jp/entry/K22906) | Organic phosphoester hydrolysis | 3-phytase |
| ppx | K01524 | Purine metabolism | Exopolyphosphatase/guanosine-5'-triphosphate,3'-diphosphate pyrophosphatase(General purine/polyphosphate metabolism gene) |
| purC | K01923 | Purine metabolism | Phosphoribosylaminoimidazole-succinocarboxamide synthase(Central purine metabolism housekeeping gene) |
| pyrG | K01937 | Pyrimidine metabolism | CTP synthase(Central pyrimidine metabolism housekeeping gene) |
| ugpA | K05814 | Transporters | Sn-glycerol 3-phosphate transport system permease |
| ugpC | K05816 | Transporters | Sn-glycerol 3-phosphate transport system ATP-binding protein |
| ugpE | K05815 | Transporters | Sn-glycerol 3-phosphate transport system permease |
| pgtA | K08476 | Two-component system | Response regulator PgtA (NtrC family) |
| phnD | K02044 | Transporters | Phosphonate transport system substrate-binding protein |
| pstS | K02040 | Transporters | Phosphate transport system substrate-binding protein |
| pit | [K03306](https://www.genome.jp/entry/K03306) | Transporters | Inorganic phosphate transporter |
| phnU | [K11083](https://www.genome.jp/entry/K11083) | Transporters | 2-aminoethylphosphonate transport system permease |
| phnT | K11084 | Transporters | 2-aminoethylphosphonate transport system ATP-binding protein |
| aepX | K01841 | Transporters | 2-aminoethylphosphonate substrate-binding protein |
| pps | K09773 | Pyruvate metabolism | Pyruvate, water dikinase(Central carbon metabolism gene) |
| nrdE | [K00525](https://www.genome.jp/entry/K00525) | Pyrimidine metabolism | Ribonucleoside-diphosphate reductase alpha chain(Central pyrimidine metabolism housekeeping gene) |
| dut | K01520 | Pyrimidine metabolism | dUTP pyrophosphatase(pyrimidine metabolism housekeeping gene) |
| purS | [K23264](https://www.genome.jp/entry/K23264) | Purine metabolism | Phosphoribosylformylglycinamidine synthase subunit PurS(Central purine metabolism housekeeping gene) |
| gmk | [K00942](https://www.genome.jp/entry/K00942) | Purine metabolism | Guanylate kinase(Central purine metabolism housekeeping gene) |
| ptsH | [K02784](https://www.genome.jp/entry/K02784) | Phosphotransferase system | Phosphocarrier protein(carbohydrate phosphotransferase system gene) |
| fomC | [K12901](https://www.genome.jp/entry/K12901) | Phosphonate and phosphinate metabolism | Phosphonoacetaldehyde reductase |
| phnM | K06162 | Phosphonate and phosphinate metabolism | Alpha-D-ribose 1-methylphosphonate 5-triphosphate diphosphatase |
| gcd | K00117 | Pentose phosphate pathway | Quinoprotein glucose dehydrogenase(Central carbohydrate metabolism gene) |
| gnd | [K00033](https://www.genome.jp/entry/K00033) | Pentose phosphate pathway | 6-phosphogluconate dehydrogenase(Central pentose-phosphate pathway gene) |
| pafA | K13571 | Organic phosphoester hydrolysis | Phosphoate-insensitive phosphomonoesterase |
| glpQ | K17323 | Organic phosphoester hydrolysis | Glycerophosphoryl diester phosphodiesterase (periplasmic) |

Table S13. TPM-normalized abundance of phosphorus-cycling functional genes across all treatments.

| Genes | S1CK | S1K5 | S1K10 | S1K15 | S3CK | S3K5 | S3K10 | S3K15 |
| --- | --- | --- | --- | --- | --- | --- | --- | --- |
| aepX | 3.849 | 3.375 | 2.750 | 3.047 | 4.531 | 2.427 | 6.658 | 4.758 |
| cmk | 1380.310 | 1338.786 | 1311.902 | 1265.372 | 1451.409 | 1431.927 | 1313.886 | 1345.428 |
| dcd | 232.345 | 239.412 | 236.777 | 245.505 | 265.994 | 236.384 | 243.688 | 254.026 |
| dut | 394.470 | 394.627 | 386.869 | 392.544 | 424.537 | 406.398 | 386.119 | 423.093 |
| fomC | 7.594 | 7.601 | 8.084 | 6.172 | 7.311 | 8.681 | 11.127 | 7.938 |
| gcd | 979.143 | 1051.969 | 1090.830 | 1004.536 | 779.806 | 765.117 | 893.982 | 931.470 |
| gdh | 343.564 | 329.208 | 323.417 | 327.648 | 285.138 | 278.245 | 297.345 | 301.221 |
| glpQ | 474.297 | 442.430 | 445.817 | 445.720 | 469.140 | 449.676 | 466.636 | 430.725 |
| gmk | 379.682 | 382.361 | 378.693 | 394.251 | 387.908 | 385.347 | 357.483 | 350.599 |
| gnd | 654.616 | 646.628 | 638.577 | 635.971 | 677.301 | 633.731 | 658.338 | 685.526 |
| gnl | 985.423 | 963.925 | 958.901 | 921.868 | 1021.716 | 1259.095 | 1356.022 | 1207.672 |
| gntK | 754.057 | 759.932 | 757.666 | 714.534 | 763.462 | 744.998 | 763.585 | 742.335 |
| guaA | 1199.769 | 1209.380 | 1181.005 | 1161.802 | 1190.631 | 1157.481 | 1154.945 | 1188.976 |
| htxB | 1.067 | 0.634 | 1.138 | 0.255 | 1.218 | 0.586 | 0.368 | 1.139 |
| mpnS | 0.102 | 0.840 | 0.042 | 0.286 | 0.772 | 0.669 | 0.988 | 0.394 |
| nrdE | 11.525 | 9.460 | 11.165 | 10.104 | 14.118 | 7.674 | 7.399 | 6.883 |
| pafA | 12.847 | 10.191 | 11.928 | 11.020 | 13.083 | 11.776 | 8.998 | 8.104 |
| pgtA | 19.132 | 23.101 | 19.929 | 19.670 | 24.768 | 34.388 | 25.875 | 22.016 |
| pgtC | 4.559 | 6.296 | 5.251 | 6.908 | 7.158 | 7.541 | 3.297 | 6.295 |
| phnD | 59.503 | 55.365 | 60.988 | 64.212 | 75.037 | 67.968 | 74.835 | 85.558 |
| phnM | 125.693 | 126.771 | 127.192 | 134.355 | 105.747 | 91.300 | 104.166 | 113.871 |
| phnP | 182.782 | 171.323 | 162.814 | 172.375 | 182.348 | 182.670 | 181.869 | 189.027 |
| phnT | 6.952 | 7.850 | 5.669 | 6.304 | 3.046 | 5.385 | 6.048 | 5.110 |
| phnU | 32.672 | 30.717 | 32.366 | 31.349 | 31.900 | 26.436 | 31.349 | 35.046 |
| phnV | 56.392 | 51.985 | 52.944 | 49.624 | 49.091 | 47.635 | 53.339 | 53.698 |
| phy | 138.450 | 117.871 | 117.709 | 115.953 | 110.382 | 123.054 | 111.080 | 106.949 |
| pit | 151.687 | 154.273 | 152.029 | 162.543 | 132.885 | 127.933 | 104.156 | 111.326 |
| pphA | 3.427 | 4.889 | 3.937 | 4.127 | 3.014 | 2.945 | 5.917 | 4.462 |
| pps | 522.137 | 514.812 | 515.856 | 515.330 | 494.660 | 461.264 | 488.311 | 535.379 |
| ppx | 228.407 | 236.403 | 239.349 | 219.046 | 223.483 | 208.222 | 207.866 | 207.117 |
| pstS | 2652.624 | 2566.474 | 2534.104 | 2496.700 | 2287.207 | 2151.852 | 2090.896 | 2069.879 |
| ptsH | 271.416 | 270.060 | 261.814 | 255.151 | 264.068 | 249.388 | 235.054 | 231.652 |
| purC | 1031.985 | 1002.410 | 978.564 | 955.728 | 1095.450 | 1046.421 | 1010.960 | 1017.085 |
| purS | 92.542 | 83.067 | 84.885 | 91.284 | 110.842 | 92.645 | 89.627 | 87.753 |
| pyrG | 880.422 | 906.684 | 914.108 | 872.512 | 867.161 | 858.363 | 824.951 | 836.525 |
| SenX3 | 178.776 | 172.843 | 172.315 | 164.351 | 163.460 | 157.126 | 151.199 | 163.902 |
| ugpA | 746.938 | 711.807 | 700.103 | 716.714 | 769.192 | 661.937 | 754.672 | 717.621 |
| ugpC | 1688.858 | 1618.264 | 1645.927 | 1653.661 | 1554.609 | 1442.169 | 1571.542 | 1565.464 |
| ugpE | 726.493 | 669.445 | 676.134 | 684.655 | 743.350 | 648.085 | 764.133 | 690.409 |

Table S14. PERMANOVA global test results based on Bray-Curtis dissimilarity, evaluating the effects of potassium application gradients on the microbial community structure in topsoil and subsoil layers.

| Topsoil | Df | SumsOfSqs | MeanSqs | F.Model | R^2^ | Pr(>F) |
| --- | --- | --- | --- | --- | --- | --- |
| Treat | 3 | 0.0134 | 0.0045 | 0.8674 | 0.2454 | 0.564 |
| Residuals | 8 | 0.041 | 0.0051 | NA | 0.7546 | NA |
| Total | 11 | 0.0544 | NA | NA | 1 | NA |
| Subsoil | Df | SumsOfSqs | MeanSqs | F.Model | R^2^ | Pr(>F) |
| Treat | 3 | 0.0653 | 0.0218 | 1.6484 | 0.382 | 0.021 |
| Residuals | 8 | 0.1057 | 0.0132 | NA | 0.618 | NA |
| Total | 11 | 0.171 | NA | NA | 1 | NA |
